# Interventions Targeting the Gut Microbiome to Improve Cancer Treatment Outcomes and Their Gastrointestinal Side Effects: A Systematic Review and Meta-analysis

**DOI:** 10.1016/j.tjnut.2025.101300

**Published:** 2025-12-29

**Authors:** Cecilia Morel, Ruijie Li, Carmen Fiuza Luces, Mark FH Brougham, Juan Francisco Pascual-Gazquez, Luciana Torquati, Raquel Revuelta Iniesta

**Affiliations:** 1 Public Health and Sport Sciences, Medical School, University of Exeter, Exeter, UK; 2 Wolfson Institute of Population Health, Queen Mary University London, London, UK; 3 Research Institute of the Hospital 12 de Octubre (imas12), Madrid, Spain; 4 Department of Paediatric Oncology and Haematology, Royal Hospital for Children and Young People, Edinburgh, UK; 5 Pediatric and Adolescent Hematology and Oncology Service, Pediatrics and Pediatric Surgery Clinical Management Unit, Virgen de las Nieves University Hospital, Granada, Spain

**Keywords:** gut microbiome interventions, gastrointestinal adverse events (GIAEs), cancer, probiotics, prebiotics, synbiotics, fecal microbiota transplantation

## Abstract

**Background:**

Improvements in cancer treatment are essential to reduce premature mortality. Emerging evidence highlights the role of the gut microbiome (GM) in influencing treatment responses and modulating gastrointestinal adverse events (GIAEs). Because cancer therapy disrupts GM composition, restoring gut health may help mitigate side effects and support gut-associated immunity.

**Objectives:**

This study aimed to systematically evaluate and assess the effectiveness of GM interventions on the occurrence of GIAEs and clinical responses to cancer treatment.

**Methods:**

Three databases (PubMed, Web of Science, and Cochrane Library) were systematically searched up to February 2025 for studies assessing GM interventions during cancer treatment. Risk of bias was evaluated using the Effective Public Healthcare Panacea Project Quality Assessment tool. Meta-analyses were conducted in Stata 18 using random-effects models to estimate the pooled relative risk of GM interventions on gut microbiome interventions, gastrointestinal adverse events (GIAEs) (primary outcome) and objective disease response rates (secondary outcome).

**Results:**

Fifty-six studies were included in the systematic review, and 40 were meta-analyzed (*n* = 37 for GIAE outcomes, *n* = 8 for treatment response). GM interventions reduced the overall risk of GIAEs [relative risk (RR)]: 0.59; 95% CI: 0.53, 0.65; *I*^2^: 76.8%; 95% prediction interval (PI): 0.32, 1.08], including diarrhea, constipation, nausea, and vomiting, but with considerable heterogeneity between studies. There was insufficient evidence to suggest improvements in objective disease response rates (RR: 1.06; 95% CI: 0.93, 1.20; *I*^2^: 0%; 95% PI: 0.93, 1.20).

**Conclusions:**

GM interventions show promise in improving cancer care by reducing GIAEs, although evidence for direct effects on-treatment response remains limited. Standardizing intervention protocols and outcome reporting in future RCTs is essential to strengthen the evidence base and guide clinical recommendations.

This trial was registered at PROSPERO as CRD42023443332 (https://www.crd.york.ac.uk/prospero/display_record.php?ID=CRD42023443332).

## Introduction

Cancer caused 10 million deaths worldwide in 2020, highlighting the need for improved treatments and supportive care [1]. Recent research suggests that the gut microbiome (GM), an ecosystem of bacteria—archaea and eukarya inhabiting our gastrointestinal tract—may influence response to cancer treatment and mitigate treatment-associated side effects [2,3].

The GM helps train the immune system, maintain immune homeostasis, and protect against infections. Approximately 70% of the body’s immune cells are located in the gut-associated lymphoid tissue, suggesting a direct connection between the GM and immune function [4]. The homeostasis of the innate and adaptive immune systems depends largely on eubiosis, where microbiota remain either commensal or symbiotic with their hosts, can compete for nutrients and produce antimicrobial compounds to prevent pathogenic infections [5,6]. When the GM suffers disruptions, such as by use of antibiotics or cancer treatment, opportunistic pathogens may grow with a respective decrease in commensal bacteria abundance (dysbiosis), potentially resulting in imbalanced immune responses [7]. Such changes may have the potential to alter the efficacy and toxicity of cancer treatments, as well as increase susceptibility to infections and inflammatory disorders [[8], [9], [10]].

Evidence suggests that probiotic bacteria may release immunomodulators and promote the secretion of anti-inflammatory factors, enabling them to act as pathogen antagonists and induce cytokine and chemokine production [11]. Probiotics are defined as live microorganisms that provide health benefits when consumed adequately and are dependent on prebiotics, which serve as their energy source [11,12]. Probiotics and prebiotics can be combined into synbiotics [13,14].

Several cancer clinical trials have demonstrated that probiotics, prebiotics (including nutritional supplements), and synbiotics can reduce gastrointestinal adverse events (GIAEs) such as diarrhea, constipation, nausea, and vomiting [[15], [16], [17], [18], [19]]. GIAEs can affect ≤90% of patients with cancer [20,21], leading to decreased treatment adherence and/or treatment delays due to dose adjustments and hospitalization [22], which can result in poorer treatment outcomes [23]. Although this field is still in its infancy, multiple studies have also attempted to manipulate GM composition with the hope of improved responses to therapy. Two studies involving patients with immunotherapy-resistant melanoma demonstrated that fecal microbiota transplantation (FMT), which transfers the entire GM from one host to another, induced objective responses [24,25], defined as a partial or complete response to the cancer treatment [26]. Similarly, objective responses were better in immunotherapy patients taking probiotics than those who did not [27,28].

Immune responses are closely tied to the GM, which interacts with the immune system via a single layer of intestinal epithelial cells that form the gut barrier [29]. The GM produces anti-inflammatory short-chain fatty acids that reduce local inflammation and enhance the gut barrier by nourishing enterocytes. Disruption to this mucosal barrier by environmental factors (such as chemotherapy) can facilitate the translocation of gut bacteria, allowing microbes to find themselves in close contact with enterocytes or the blood stream, activating proinflammatory pathways and dampening immune responses [30]. On the contrary, stimulation of short-chain fatty acid production via diet or probiotic supplementation can stimulate antibody production and immune cell expansion and decrease systemic inflammation [11]. This can help maintain the integrity of the intestinal barrier, potentially mitigating gastrointestinal toxicity [11,29].

Currently, clinical guidelines on GM interventions in cancer are lacking, with concerns about the use of probiotics in immunocompromised patients [31]. Although previous meta-analyses have explored the impact of probiotics on chemotherapy and radiotherapy-induced side effects, these reviews often yield inconsistent results and do not address disease responses [[32], [33], [34], [35], [36]]. With the growing body of research on the GM’s role in cancer, more comprehensive systematic reviews are needed to guide clinical practice [37]. This systematic review aimed to summarize, evaluate, and quantitatively assess the impact of GM-targeting interventions on responses to cancer treatment and GIAEs.

## Methods

### Literature search and identification of studies

This study was performed following the Population, Intervention, Comparator, Outcomes and Study design (PICOS) model [38], which adheres to the PRISMA [39]. The protocol of this systematic review has been registered (registration number: CRD42023443332) with the PROSPERO. This study has deviated from its protocol in terms of primary outcome (changed from disease response to GIAEs because of a limited number of studies reporting response). Relevant studies were identified by a systematic search of 3 electronic databases (CM): PubMed, Cochrane CENTRAL Register of Controlled Trials, and Web of Science, from inception to February 2025, and additional studies identified through backward reference tracing (screening of reference lists of included studies and relevant systematic reviews). An initial search strategy was developed in PubMed and modified for the other databases. The following keywords and their synonyms were used as search terms: (“cancer” OR “neoplasm” OR “tumour”) AND (“chemotherapy” OR “radiotherapy” OR “immunotherapy”) AND (“gut microbiome” OR “probiotic” OR “prebiotic” OR “synbiotic” OR “faecal microbiota transplant” OR “dietary fibre”). See Supplemental Material 1 for full list of search terms.

### Research question and outcomes

The research question was “In cancer patients undergoing treatment (P), how does an intervention on the GM (I) affect the incidence of gastrointestinal adverse events (primary outcome) and objective disease response rate (secondary outcome) (O) compared to control or base rate (C)?" The primary outcome was incidence of GIAEs (side effects), and the secondary outcome was objective disease response rate, and safety was to be reported in terms of death or serious adverse events.

### Eligibility criteria

The studies included in the systematic review were screened following the principle of PICOS—population: studies that concern patients with cancer undergoing treatment; intervention: any use of diet, pro/pre/synbiotic supplementation, or any other treatment aimed to manipulate GM composition and/or function in addition to cancer treatment; comparison: no intervention (baseline) or placebo or specific control condition; outcomes: adverse events must be reported; and studies: no language restrictions on studies.

Studies were excluded if any of the following reasons were involved: interventions exclusively investigating the negative effects of drugs (e.g., antibiotics disrupting the GM), without the addition of pro/pre/synbiotics, FMT, or a specific diet aimed at manipulating the GM; ongoing or unpublished experiments; observational studies, case studies, case-control studies, reviews, retrospective articles, animal experiments, independent protocols, letters, books, and personal opinions.

### Screening and data extraction

Titles and full texts were screened and selected (CM and RL) and any disagreements were solved by RRI. Requisite data was extracted independently by 2 reviewers (CM and RL) into a data extraction form. The extracted data contained: first author, publication year, country, characteristics of participants (cancer type, age, and sex), treatment regimen, sample size, intervention, dose and length of intervention, details of comparator, outcomes, outcome reporting methods, adverse events/safety, and study design.

### Risk of bias assessment

The Effective Public Healthcare Panacea Project Quality Assessment tool for Quantitative Studies was used to assess the quality of each study by 2 reviewers (CM and RL) and in case of disagreement, a third reviewer (LT) made the final decision [40]. Each study was allocated a strong, moderate, or weak quality rating to assess methodologic quality (data collection practices and withdrawals and dropout), appropriateness of study design, and risk of bias (selection bias, confounders, and blinding). All included studies were assessed for bias, before screening for meta-analysis eligibility.

### Data analysis and meta-analysis

Studies were screened after data extraction and included in the meta-analyses if they met the following criteria: *1*) the primary outcome (gastrointestinal adverse events) and/or secondary outcome (objective response) were reported as binary counts (2 × 2 event counts); and *2*) the study followed the principle of a controlled clinical trial. Eligible studies that were not included in the meta-analysis were described narratively using Synthesis Without Meta-analysis guidelines [41]. Meta-analyses were performed using the DerSimonian and Laird random-effects model to estimate the effect size of GM interventions on GIAEs and objective response rates (ORRs), including the pooled relative risk (RR), 95% CIs, and 95% prediction intervals (PI). Heterogeneity between trials was assessed by using the *I*^2^ statistic, considering a substantial level of heterogeneity when the *I*^2^ ≥ 50% [42]. Publication bias was assessed using funnel plot asymmetry testing and the modified version of Egger regression test, the Harbord test, which better accounts for between-study heterogeneity—a *P* value of 0.1 was used for significance, as is common for such tests in meta-analyses [43,44]. All statistical analyses were conducted in Stata version 18.0 Standard Edition.

For the meta-analysis, diarrhea, constipation, nausea, and vomiting were defined as occurrence of ≥1 episode. Response to treatment was defined when authors reported ORRs—objective responses were defined as a partial or complete response to the cancer treatment; a partial response is a decrease in the size of a tumor or in the amount of cancer in the body, and a complete response is the disappearance of all signs of cancer [26]. Subgroup analyses were performed if there were ≥3 studies using the same treatment methodology or reporting the same outcome. For GIAEs, sensitivity analyses were conducted on adults, children, and RCTs.

## Results

### Study selection

**FIGURE 1:**
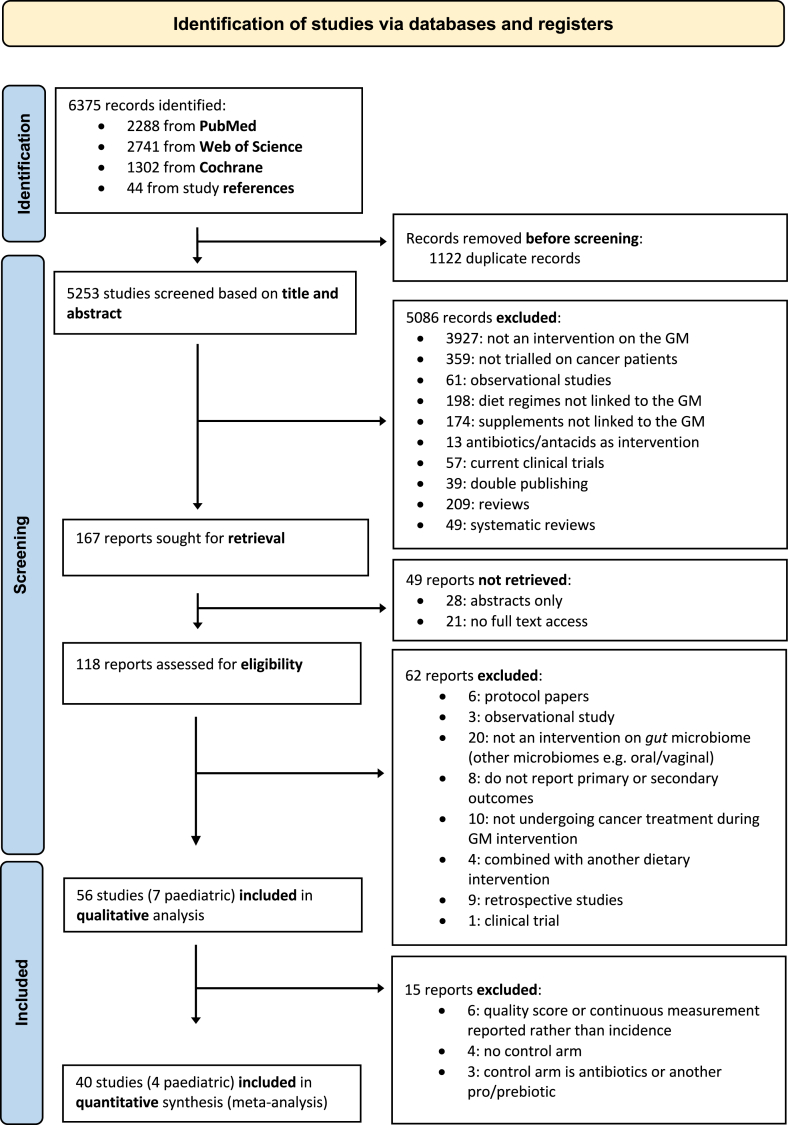
PRISMA flow chart. GM, gut microbiome.

A total of 6375 articles were retrieved and the screening procedure (Figure 1) identified 56 studies [[15], [16], [17], [18], [19],^24^,^25^,^27^,^28^,[45], [46], [47], [48], [49], [50], [51], [52], [53], [54], [55], [56], [57], [58], [59], [60], [61], [62], [63], [64], [65], [66], [67], [68], [69], [70], [71], [72], [73], [74], [75], [76], [77], [78], [79], [80], [81], [82], [83], [84], [85], [86], [87], [88], [89], [90], [91]] for the systematic review, of which 40 [[15], [16], [17], [18],^27^,^28^,[45], [46], [47], [48],[50], [51], [52], [53], [54], [55], [56], [57],[60], [61], [62], [63], [64], [65], [66], [67], [68],^72^,^73^,^75^,^76^,^79^,[81], [82], [83],^85^,^86^,^88^,^89^,^91^] were eligible for the meta-analysis.

### Study characteristics and population

The characteristics of the included studies are shown in TABLE 1, TABLE 2, TABLE 3, TABLE 4. A total of 4702 participants were included across all 56 studies; sample size ranged from 10 [24] to 482 [52] participants. The median age of the participants ranged from 4 to 66 y, with 7 studies being conducted in children. The country with the most studies included was China [[15], [16], [17],^48^,^55^,^62^,^63^,^79^,^81^,^85^,^86^], with most other studies published in Italy [49,51,52,61,77], Iran [19,56,59,64,66], the United States [25,27,54,74,82] and Japan [18,57,69,70]. In terms of language, 1 study was published in Chinese [79], [1] in Farsi [59]—all others were published in English; 36% of interventions included patients diagnosed with cervical [47,50,52,60,65,[75], [76], [77],^80^,^83^,^89^,^90^] or colorectal cancer [15,19,67,68,72,79,89,90], and chemotherapy was most commonly used (20 studies) [[15], [16], [17], [18], [19],^46^,^49^,^56^,^59^,^63^,^64^,[67], [68], [69], [70],^73^,^78^,^79^,^81^,^85^,^88^]. Over half (51.7%) of the studies used probiotics as their intervention [15,16,27,28,45,47,48,[51], [52], [53], [54], [55],^60^,^62^,^63^,[65], [66], [67], [68],^72^,^75^,^78^,^79^,^81^,^82^,^85^,^86^,^88^,^90^]. Of 56 included studies, 12 used prebiotic supplementation (4 being high/soluble fiber diets) [18,46,49,58,73,76,80,83,84,87,89,91]; 11 used synbiotic supplementation [17,19,50,56,57,59,64,[69], [70], [71],^77^]; and FMT was used in 4 studies [24,25,61,74].

**TABLE 1:** Characteristics of probiotic studies

Study	Country	Malignancy	Cancer treatment regimen	Participants (treatment arm, control arm)	Intervention	Dose and length of intervention	Comparator	GIAE and response outcomes	Outcome reporting method	Study design	Study quality rating
Radiotherapy (RT)											
Delia et al. [51], 2002	Italy	Pelvic cancers	Total dose: 60–70 Gy, daily dose: 180 cGy for 6–7 wk	*N:* 190 (95, 95) 49% F 45–65 y	Probiotic: VSL#3 bag: 7 strains of bacteria	- 10^12^ bacteria/g of VSL#3 - 1 bag 3×/d - On empty stomach, from day 1 of RT	No probiotic treatment	Grade 3/4 diarrhea frequency ↓ in probiotic group: • Grade 1: 9.5% (probiotic) vs. 9.5% (placebo) • Grade 2: 21.1% vs. 21.1% • Grade 3: 7.4% vs. 16.8% • Grade 4: 0% vs. 12.6%	WHO grading	Non-RCT	Weak
Delia et al. [52], 2007	Italy	Sigmoid, rectal, or cervical cancers	Postoperative; total dose: 60–70 Gy	*N:* 482 (243, 239)	Probiotic: VSL#3 sachet: *L. casei* *L. plantarum* *L. acidophilus* *L. delbruekii* *B. longum* *B. breve* *B. infantis* *S. salivarius* subsp. *thermophilus*	- 4.5 × 10^11^/g viable bacteria per sachet - 1 sachet 3×/d - From day 1 to end of RT	Placebo: VSL#3-identical appearing placebo	Grade 3/4 diarrhea frequency ↓ in probiotic group: • Grade 3/4 diarrhea: 3.3% (probiotic) vs. 28.9% (placebo); *P* < 0.001 • Grade 1–4 diarrhea: 17.3% vs. 49.8% • NS Δgrade 1–2 diarrhea	WHO grading	RCT, bb	Moderate
Du et al. [55], 2018	China	Central nervous system tumors: glioblastoma, medulloblastoma, ependymocytoma, and astrocytoma	Craniospinal irradiation started 2–4 wk postsurgery: 36 Gy (21–54 Gy), posterior fossa boost: 1.5 Gy (1.5–1.8 Gy)	*N:* 160 (80, 80) 33% F 1.3–15.5 y	Probiotic: *Bacillus licheniformis*	- 1 capsule, 3×/d - From 1 d before RT until end of RT	Placebo	GIAE frequency ↓ in probiotic group: • Diarrhea (any): 8.9% (probiotic) vs. 25% (control); *P* = 0.006 • Diarrhea (≥grade 3): 3.8% vs. 10%; *P* = 0.006 • Nausea: 66.3% vs. 82.5%; *P* = 0.019 • Vomiting: 35.0% vs. 52.5%; *P* = 0.026 ORR ↑ in probiotic group vs. control: • PR: 16.3% vs. 17.5% • CR: 25.0% vs. 18.8%	National Cancer Institute CTCAE v3.0. CR: Full disappearance of visible tumor. PR: 50% reduction in tumor volume	Non-RCT	Moderate
Mansouri-Tehrani et al. [66], 2016	Iran	Pelvic cancers	Total dose: 4000–5000 cGy, daily dose: 1.8 Gy, 18 MV, 5 fractions/wk for 4–5 wk	*N:* 67 (43, 24) 42% F 20–85 y	Probiotic: LactoCareÒ capsule (500 mg): *L. casei*: 1.5×10^9^ CFU *L. acidophilus*: 1.5 × 10^10^ *L. rhamnosus*: 3.5 × 10^9^ *L. bulgaricus*: 2.5 × 10 *B. breve*: 1 × 10^10^ *B. longum*: 5 × 10^8^ *S. thermophilus*: 1.5 × 10^8^	- Group 1: 2 capsules/d with 150 g low-fat yogurt - Group 2: 1 capsule + 15 g honey (2×/d morning/evening) with 150 g yogurt - Duration: 1 wk before RT, continued for 5 wk	Placebo: 500 mg corn starch	Diarrhea grade ↓ in intervention groups: • Grade 2/3: 31.8% (group 1) vs. 19.0% (group 2) vs. 70.8% (placebo) • Week 5 mean grade: 1 (group 1) vs. 2 (placebo); *P* = 0.001 • Week 5 mean grade: 1 (group 2) vs. 2 (placebo); *P* <0.001	CTCAE v2.0 and Bristol Stool Scale	RCT	Strong
Salminen et al. [75], 1988	Finland	Cervix or uterus carcinoma	Internal and external RT: Intracavitary cesium, followed by Wertheim hysterectomy (1–2 wk later). After 4–6 wk, 4400 cGy in 22 fractions with a 2-wk break. Total radiation: 8000 cGy (tumor), 5000 cGy (pelvis)	*N:* 21 (11, 10) 100% F 40–75 y	Probiotic: *L*. *acidophilus* bacteria	- Daily dose of ≥2 × 10^9^ *L. acidophilus* in 150 mL yogurt - Taken 5 d before RT, daily during RT (including interval), and 10 d post-RT	No probiotic treatment	Diarrhea frequency ↓ in probiotic group: • Week 5 incidence: 27.3% (probiotic) vs. 90.0% (control); *P* < 0.01	Self-reported via physician interviews	RCT	Moderate
Chemoradiotherapy)											
Ahrén et al. [45], 2023	Sweden	Gynecologic Cancer	External beam RT (≥40 Gy) to the pelvis, with or without chemotherapy, for 23–36 d	*N:* 97 (50, 25) 100% F Mean: 56 y	Probiotic: *L*. *plantarum* HEAL9 and *L*. *plantarum* 299	- Group 1: low-dose probiotic (LDP, 1 × 10^10^ CFU/capsule) - Group 2: high-dose probiotic (HDP, 5 × 10^10^ CFU/ capsule). - 2 capsules/d - Taken 1–2 wk before RT until 2 wk posttreatment	Placebo	Defecation urgency and nausea ↓ in probiotic groups: • Loose stools: 1.06 ± 0.75 (HDP) vs. 1.40 ± 1.05 (placebo); *P* = 0.3 • Defecation urgency (d): 29% ± 18% (LDP) vs. 61% ± 32% (placebo); *P* = 0.042 • Nausea days ↓ in LDP vs. placebo; *P* = 0.06	Self-reported via participants daily gastrointestinal health diary and a quality of life questionnaire	RCT, bb	Strong
Chitapanarux et al. [47], 2010	Thailand	Cervical squamous cell carcinoma	External pelvic RT (200 cGy per fraction; total: 5600 cGy) plus cisplatin (40 mg/m^2^ weekly for 6 wk); 5 fractions per week; 4 brachytherapy insertions with Iridium-192 (700 cGy per fraction)	*N:* 63 (32, 31) 100% F Median, 49.5 y	Probiotic: *L*. *acidophilus* plus *Bifidobacterium bifidum* (Infloran)	- 1 capsule (250 mg) contains 10^9^ *L. acidophilus* and 10^9^ *B. bifidum* - 2 capsules 2×/d before meals (morning and evening) - Start 7 d before RT and continue daily during treatment	Placebo capsules: magnesium stearate, talc, and purified water	Severe diarrhea ↓ in probiotic vs. placebo group (*P* = 0.002): • Grade 1: 90.6% (probiotic) vs. 54.8% (placebo) • Grade 2–3: 9.4% vs. 45.2% • All grades: 100.0% vs. 100.0%	CTCAE v2.0	RCT, bb	Strong
Demers et al. [53], 2014	Canada	Pelvic (prostatic, gynecologic, and rectal) cancers	External pelvic RT: 1.8–2.0 Gy daily, 5 d/wk, total 40–50.4 Gy. Chemotherapy (cisplatin 40 mg/m^2^ or 5-FU 225 mg/m^2^) during RT. Some patients received brachytherapy before or aftertreatment.	*N:* 229 (14, 89) 33% F Mean 61 y	Probiotic: *L. acidophilus* LAC-361 and *B. longum* BB-536 (Bifilact)	- Group 1: Standard dose group (SDG): 1.3 × 10^9^ CFU, 2×/d - Group 2: High-dose group (HDG): 10^10^ CFU, 3×/d - Start on the first day and end on the last day of RT	Placebo	Diarrhea ↓ in probiotic groups: • Grade 1: 17% (HDG) vs. 22% (SDG) vs. 14% (control) • Grade 2: 42% vs. 46% vs. 49% • Grade 3: 17% vs. 15% vs. 20% • Grade 4: 7% vs. 3% vs. 11% • *P* = 0.05 for patients with pelvic surgery; *P* = NS for without	WHO grading and Bristol Stool Scale.	RCT, bb	Strong
Giralt et al. [60], 2008	Spain	Cervical carcinoma or endometrial adenocarcinoma	Pelvic RT with linear accelerator (15–18 MV): 1.8–2 Gy daily, total 45–50.4 Gy, 5 times/wk for 5–6 wk. Patients with cervical cancer received cisplatin 40 mg/m^2^ weekly during RT. Brachytherapy given 2–3 wk later	*N:* 85 (44, 41) 100% F Mean 60 y	Probiotic: *L. casei* DN-114 001, *S thermophiles*, and *L*. *delbrueckiim* subsp. *bulgaricus*	- 96 mL 3×/d of fermented liquid yogurt - ≈10^8^ CFU/g *L. casei* DN-114 001 + standard starters *S. thermophilus* and *L. delbrueckii* subsp. *bulgaricus* - 1-wk run-in period before RT, continued daily during treatment	Placebo	NS Δ in diarrhea frequency: • ≥Grade 3 diarrhea: 45% (probiotic) vs. 37% (control); *P* = NS • Time to ≥grade 2 diarrhea: 44 vs. 41 d • First type 6 stool onset: 14 vs. 10 d; *P* = 0.048 NS Δ in QoL scores	CTCAE v2.0, Bristol Stool Scale, EORTC-QLQ-C30	RCT, bb	Strong
Jiang et al. [62], 2019	China	Nasopharyngeal Carcinoma	RT with concurrent cisplatin: 2.19 Gy daily, 5 d/wk, total 70 Gy in 32 fractions. Cisplatin (100 mg/m^2^) on days 1, 22, and 43	*N:* 93 (58, 35) 38% F Med 51 y	Probiotic: *B. longum*, *L. lactis*, and *E. faecium*	- 3 capsules, 2×/d, for ≤7 wk	Placebo	NS Δ in ORR: • CR: 87.9% (probiotic) vs. 88.6% (control) • PR: 12.1% vs. 11.4% (*P* > 0.999)	Response Evaluation Criteria in Solid Tumors (RECIST)	RCT, bb	Strong
Linn et al. [65], 2019	Myanmar	Cervical cancer	Pelvic RT, 50 Gy with or without concurrent chemotherapy. 5 fractions/wk for 5 wk	*N:* 54 (26, 28) 100% F Mean 55 y	Probiotic: *L. acidophilus* LA-5 and *B. animalis* subsp. *lactis* BB-12 (Biogurt)	- Each capsule: 300 mg functional yogurt with 1.75 × 10^9^ *L. acidophilus* and *B. lactis* - 1 capsule taken 3×/d - Starting on the first day of RT and continuing until end of RT	Placebo	Diarrhea ↓ in probiotic group: • Grade 1–2: 50.0% (probiotic) vs. 79.3% (control) (*P* = 0.025) • Grade 3–4: 0% vs. 17.2% (*P* = 0.05)	CTCAE v4.0	RCT, bb	Strong
Liu et al. [86], 2024	China	Hepatocellular carcinoma	Transhepatic arterial chemotherapy and embolization: oxaliplatin (100 mg/m^2^) + epirubicin (30 mg/m^2^) + 5–10 mL lipiodol (40%), every 3–4 wk for 2–3 cycles. Intensity-modulated RT: 5–8 radiation fields (6-MV X-rays), 2 Gy/session, 5×/wk for 4–6 wk (total 50–60 Gy)	*N:* 92 (46, 46) 33.7% F Mean 64.7 ± 6.4 y	Probiotic: *Bifidobacterium* and *Lactobacillus* species	- 2–4 oral live capsules 2×/d during RT	No probiotic treatment	ORR ↑ in probiotic group vs. control • ORR: 84.8% vs. 65.2%; *P* < 0.05 • CR: 37.0% (probiotic) vs. 28.2% (control) • PR: 47.8% vs. 37.0%	CR: Full lesion disappearance ≥4 wk PR: >50% lesion reduction ≥4 wk Nonresponse: No change or lesion growth ORR: CR + PR	RCT	Weak
Osterlund et al. [72], 2007	Finland	Colorectal cancer	Adjuvant chemotherapy postsurgery: monthly 5-FU + leucovorin (Mayo regimen) or bimonthly 5-FU bolus + infusion (simplified de Gramont) for 24 wk. Some patients with rectal cancer received RT.	*N:* 148 (97, 51) 50% F 31–75 y	Probiotic: *L. rhamnosus* GG -One-third patients supplemented with Guar gum nutritional supplement (fiber)	- *L. rhamnosus*: 1–2 × 10^10^ CFU/d - 1 capsule taken 2×/d for 24 wk of chemotherapy - Capsules swallowed or dissolved in cold milk/juice - 500 mL supplement containing 11 g guar gum taken daily on cycle days 7–14, for 8 d/mo - All patients received dietary counseling	No probiotic treatment, dietary counseling	Grade ≥ 3 diarrhea ↓ in probiotic group • Grade 0–2: 78% (probiotic) vs. 63% (control) • Grade 3–4: 22% vs. 37%; OR: 0.38 (95% CI: 0.16, 0.89; *P* = 0.027) Addition of fiber: NS Δ in GIAEs (*P* = 0.13).	CTCAE v2.0	RCT	Moderate
Timko [90], 2010	Slovakia	Colorectal, rectosigmoid junction, uterine, urinary bladder, cervical, anus, anal canal, prostate, and sigmoid colon cancer	RT: Cobalt-60, 4-field box technique. Standard dose: 50 Gy (2 Gy/d, 5–7 wk) High-risk patients: 65–67 Gy (2 Gy/d). 52.5% of patients received 500 mg 5-FU for 1 wk during RT	*N:* 42 (22, 20) 33.3% F Med. 64.5 (34–83) y	Probiotic: “5” Strain Dophilus containing: *L. rhamnosus* (55%) *B. adolescentis* (20%) *L. acidophilus* (5%) *B. longum* (5%) *E. faecium* (15%)	- 1 capsule, 2×/d - 6 × 10^9^ CFU/capsule - From start of RT until end of RT (5–7 wk)	Hylak Tropfen Forte (cell-free fermentation products of *L. helveticus* and gut symbionts)—40 drops taken 3×/d	Diarrhea ↓ in probiotic group: • Liquid stools: 4% (probiotic) vs. 22% (control) Daily number of bowel movements ↑ in probiotic group: • Mean: 4.16 (1.2–9.7) vs. 2.52 (0.7–5.3)	Self-reported by patient	RCT	Weak
Chemotherapy)											
Huang et al. [15], 2023	China	Colorectal cancer	Capecitabine and oxaliplatin, XELOX regimen after surgical resection treatment	*N:* 100 (50, 50) 47% F, Mean 59.9 ± 11.2 y	Probiotic: *B. infants*, *L. acidophilus*, *E. faecalis*, and *B. cereus*	- 0.5 g tablets contain >0.5 × 10^6^ CFU of *B. infants*, *L. acidophilus*, *E. faecalis*, and >0.5 × 10^5^ CFU of *B. cereus* - 1 tablet taken 3×/d - From third postoperative day until end of first chemotherapy course (∼ 6 wk, including 2 wk of chemotherapy)	Placebo tablets without any live probiotics	GIAEs ↓ in probiotic group: • Diarrhea: 16% (probiotic) vs. 40% (control); *P* = 0.008 • Constipation: 8% vs. 28%; *P* = 0.019 • Nausea: 22% vs. 34%; *P* = 0.181	Self-reported by patient	RCT, b	Strong
Juan et al. [63], 2022	China	Breast cancer	Up-front surgery and then standard chemotherapy: epirubicin + cyclophosphamidum, epirubicin + cyclophosphamidum combined with docetaxel or docetaxel + cyclophosphamidum	*N:* 159 (80 79) 100% F Mean 45.8 y	Probiotic: *B. longum*, *L. acidophilus*, and *E. faecalis*	- 0.84 g capsules contain *B. longum* (1.0 × 10^7^ CFU/210 mg), *L. acidophilus* (1.0 × 10^7^ CFU/210 mg), and *E. faecalis* (1.0 × 10^7^ CFU/210 mg) - 3 capsules taken 2×/d during chemotherapy	Placebo capsules contain same ingredients except probiotics	Constipation ↓ in probiotic group (*P* < 0.01) • During eighth cycle: 10% (probiotic) vs. 75% (control); *P* < 0.01	No information	RCT, bb	Strong
Juan et al. [85], 2025	China	Breast cancer	Pegylated liposomal doxorubicin (PLD)-based adjuvant chemotherapy: 4 cycles: PLD (30 mg/m^2^) + cyclophosphamide (600 mg/m^2^) ±4 cycles of docetaxel (100 mg/m^2^)	*N:* 155 (77, 78) 100% F No age information	Probiotic: *B. longum*, *L. acidophilus* and *E*. *faecalis*	-0.84 g capsules contain *B. longum* (1.0 × 10^7^ CFU/210 mg), *L. acidophilus* (1.0 × 10^7^ CFU/210 mg), and *E. faecalis* (1.0 × 10^7^ CFU/210 mg) -3 capsules taken 2×/d during chemotherapy	Placebo capsules contain same ingredients except probiotics	Constipation and vomiting ↓ in probiotic group (*P* < 0.001): • Constipation: 15.6% (probiotic) vs. 77% (placebo) • Vomiting: 32.5% vs. 71.8% NS Δ in diarrhea frequency: • Diarrhea: 1% vs. 5% Overall QoL scores ↑ in probiotic group (*P* < 0.001): • Diarrhea: 1.21 (probiotic) vs. 1.11 • Constipation: 1.51 vs. 1.34 • Nausea/vomiting: 1.35 vs. 1.32 (*P* = NS for individual scores)	European Organization for Research and Treatment of Cancer quality-of-life Questionnaire C30 (EORTC QLQ-C30) V 3.0 for GIAE score No information for GIAE incidence (%) criteria	RCT, bb	Strong
Mego et al. [67], 2015	Slovakia	Colorectal cancer	New line of chemotherapy: irinotecan weekly (for 28 patients) or every 2 or 3 wk (for 18 patients). Some patients had 5-FU or capecitabine. Some patients had concurrent biological therapy: cetuximab (9), bevacizumab (13).	*N:* 46 (23, 23) 43% F 42–81 y	Probiotic: *B*. *breve*, *B. bifidum*, *B. longum*, *L. rhamnosus*, *L. acidophilus*, L. cas HA-108, *L. plantarum*, *S*. *thermopilus*, *L. brevis*, and *B. infantis*	- Each capsule contains 10 × 10^9^ CFU of bacteria, including *B. breve* (25%), *B. bifidum* (20%), *B. longum* (14.5%), *L. rhamnosus* (8%), and others - Additives: inulin (40%), maltodextrin (31.4%), magnesium stearate (3%), ascorbic acid (0.4%) - One capsule taken 3×/d after meals/snacks for 12 wk	Placebo	NS Δ in diarrhea frequency: • Any diarrhea: 39.1% (probiotic) vs. 60.9% (control); *P* = 0.24 • Grade ≥ 3 diarrhea: 0% vs. 17.4%; *P* = 0.11	CTCAE v4.0	RCT, bb	Strong
Mego et al. [68], 2023	Slovakia	Colorectal cancer	Irinotecan-based chemotherapy and 5-FU-based chemotherapy, capecitabine, anti-EGFR, and anti-VEGF therapy	*N:* 233 (114, 119) 40% F 29–82 y	Probiotic: *Bifidobacterium* species BB-12 and *L. rhamnosus* LGG (Probio-Tec BG-Vcap-6.5)	- Each capsule contains 2.7 × 10^9^ CFU of *Bifidobacterium* BB-12 (50%) and *L. rhamnosus* LGG (50%) - 3 capsules/d for 6 wk during chemotherapy	Placebo: maltodextrin	NS Δ in diarrhea frequency (*P* = 0.51) • Grade 1: 14.9% (probiotic) vs. 21.8% (control) • Grade 2: 19.3% vs. 12.6% • Grade 3: 7.9% vs. 10.9% • Grade 4: 0% vs. 0.8% (*P* = 0.38 for ≥ grade 3)	CTCAE v4.0	RCT, bb	Strong
Reyna-Figueroa et al. [88], 2019	Mexico	Acute leukemia	Prednisone (60 mg/m^2^) orally, days 0–28, vincristine (2 mg/m^2^) IV, days 0, 7, 14, 21, 28, daunorubicin (30 mg/m^2^) IV, days 0, 14, *L-asparaginase* (10,000 UI/m^2^) IM, days 5, 8, 12, 15, 19, and 22	*N:* 60 (30, 30) 36.7% F Mean 10.7 y	Probiotic: *L*. *rhamnosus* GG	- 5 × 10^9^ CFU/sachet - 1 sachet 2×/d, orally for 7 d - Discontinued after 7-d course, completion of chemotherapy, or onset of neutropenia - Administered during hospitalization by medical staff	No probiotic treatment	GIAEs ↓ in probiotic group • Diarrhea: 0% (probiotic) vs. 5% (control); RR: 0.5 (95% CI: 0.2, 1.2; *P* = 0.04) • Constipation: 10% vs. 30%; RR: 0.4 (95% CI: 0.2, 0.6; *P* < 0.05) • Nausea: 10% vs. 30%; RR: 0.5 (95% CI: 0.4, 0.8; *P* = 0.04) • Vomiting: 7% vs. 20%; RR: 0.4 (95% CI: 0.2, 0.9; *P* = 0.04)	Bristol Stool Scale, physician self-reported	RCT, b	Moderate
Tian et al. [16], 2019	China	Lung cancer	Platinum-based combination chemotherapy every 3 wk	*N:* 41 (20, 21) 27% F Mean 55.5±8.55 y	Probiotic: *C*. *butyricum*	- 3 tablets (420 mg), 3×/d - Started day before first chemotherapy course and continued for 3 wk	Placebo	Diarrhea ↓ in probiotic group (15% vs. 62%; *P* = 0.017) • Grade 1: 20% (probiotic) vs. 43% (control) • Grade 2: 5% vs. 14% • Grade 3: 0% vs. 5% NS Δ in nausea and vomiting • Nausea: Grade 1: 60% vs. 38%, Grade 2: 40% vs. 62%, Grade 3: 0% • Vomiting: Grade 1: 15% vs. 10%, Grade 2: 75% vs. 67%, Grade 3: 10% vs. 24%	CTCAE v4.0	RCT, bb	Strong
Wada et al. [78], 2010	Japan	Acute lymphoblastic leukemia, acute myeloid leukemia and non-Hodgkin lymphoma, Yolk sac tumor, Ewing sarcoma, Hodgkin disease, primitive neuroectodermal tumor, and leiomyosarcoma	Chemotherapy	*N:* 42 (19, 23) 57% F 1.2 - 13.2 y	Probiotic: *B. breve* strain Yakult (BBG-01)	- 10^9^ freeze-dried BBG-01 per 1 g dose - Taken 3×/d from 2 wk before chemotherapy for 6 wk or until WBC >1000/μL and discharged	Placebo	Days with diarrhea ↓ in probiotic group: • 1.06 ± 1.73 (probiotic) vs. 2.45 ± 4.40 (placebo); *P* = 0.09 NS Δ in number of diarrhea episodes: 0.39 ± 0.50 vs. 0.55 ± 0.80; *P* = 0.23	Self-reported by physicians	RCT, b	Strong
Wang et al. [79], 2021 (Translated from Chinese)	China	Colorectal cancer	FOLFOX4 chemotherapy began within 14 d postsurgery based on patient recovery. Day 1: oxaliplatin (85 mg/m^2^ IV, 120 min). Days 1–2: Calcium folinate (200 mg/m^2^ IV, 120 min), then 5-FU: 400 mg/m^2^ IV bolus, 600 mg/m^2^ IV infusion (4 h)	*N:* 92 (46, 46) 42% F Mean 44 y	Probiotic: *C*. *butyricum*	- 700 mg *C. butyricum* per dose - 3 tablets/d (350 mg each, (≥1.5 × 10^7^ CFU/g) - Taken for 8 wk in 2-wk cycles, starting on day 1 of chemotherapy	No probiotic treatment	Nausea/vomiting ↓ in probiotic group: • 21.7% (probiotic) vs. 45.7% (control); *P* = 0.015.	No information	RCT	Moderate
Zeng et al. [81], 2024	China	Breast cancer	All patients received 4 cycles of epirubicin (100 mg/m^2^) + cyclophosphamide (600 mg/m^2^). Before chemotherapy, they received tropisetron (5 mg IV) + metoclopramide (10 mg IM)	*N:* 36 (17, 19) 100% F Mean 53 y ± 9.82 y	Probiotic: *B*. *fragilis* 839 (BF839)	- 10^6^ CFU per BF839 pack - 2 packs/d for 4 chemotherapy cycles (21 d/cycle) - tarted on the first day of chemotherapy	Placebo: maltodextrin	Grade ≥ 3 GIAEs ↓ in probiotic vs. control group: • Diarrhea: 15% (probiotic) vs. 30% (control); *P* = 0.023 • Nausea: 35% vs. 71.3%; *P* = 0.001 • Vomiting: 20% vs. 45%; *P* = 0.001	CTCAE v3.0	RCT, bb	Strong
Immunotherapy)											
Dizman et al. [27], 2022	USA	Metastatic renal cell carcinoma	Combined immunotherapy: nivolumab (3 mg/kg IV) + ipilimumab (1 mg/kg IV) every 3 wk for 12 wk, followed by nivolumab (480 mg IV) monthly	*N:* 29 (19, 10) 42% F 45–90 y	Probiotic: *C*. *butyricum* (CBM588)	- 80 mg (2.0 × 10^8^ CFU *C. butyricum* per 40 mg sachet missed in 8 oz water) taken orally 2×/d - Taken for 12 wk during nivolumab + ipilimumab treatment	No probiotic treatment	Diarrhea ↓ in probiotic group vs. control: • Any: 10.5% (probiotic) vs. 10% (control) • Grade 3–4: 5% vs. 10% ORR and PFS ↑ in probiotic group: • PFS: 12.7 mo (probiotic) vs. 2.5 mo (control) (HR: 0.15; *P* < 0.001). • ORR: 58% vs.: 20%; *P* = 0.06 • PR: 57.9% vs. 20% • Disease control: 79% vs. 40% • Tumor shrinkage: 74% vs. 50%	RECIST v1.1 criteria. Safety assessment by adverse event grading.	RCT	Moderate
Ebrahimi et al. [82], 2024	USA	Metastatic renal cell carcinoma	Immunotherapy and biological therapy: cabozantinib (40 mg) by mouth daily along with nivolumab (480 mg) once a month by IV infusion, all patients previously untreated	*N:* 30 (20, 10) 33% F 36–84 y	Probiotic: *C*. *butyricum* (CBM588)	- 80 mg (2.0 × 10^8^ CFU *C. butyricum* per 40 mg sachet) taken orally 2×/d - Taken until protocol completion, unacceptable adverse events, consent withdrawal, or disease progression	No probiotic treatment	NS Δ in GIAEs between groups: • Diarrhea: 50% (probiotic) vs. 40% (control) • Grade ≥3 diarrhea: 10% vs. 0% • Constipation: 5% (probiotic) vs. 30% (control) • Vomiting: 10% vs. 10% • Nausea: 30% vs. 10% ORR ↑ in probiotic group vs. control (74% vs. 20%; *P* = 0.01): • Target lesion size ↓ in 89% (probiotic) vs. 80% (control) • Clinical benefit (CR/PR/SD ≥ 6 mo) in 80% (probiotic) vs. 60% (control)	RECIST criteria GIAE criteria not reported	RCT	Moderate
Spreafico et al. [28], 2023	Canada	Advanced solid malignancies: HNSCC, melanoma, urothelial tract/bladder cancer, and renal cell carcinoma	Immune checkpoint inhibitors: anti–PD-1 monotherapy (nivolumab 480 mg 4 times a week, pembrolizumab 200 mg 3 times a week) or anti–PD-1 + anti–CTLA-4 (nivolumab 360 mg 3 times a week + ipilimumab 3 mg/kg or 1 mg/kg 3 times a week for 4 infusions, followed by nivolumab 480 mg 4 times a week maintenance)	*N:* 39 (29, 10) 33% F 25–84 y	Probiotic: Microbial Ecosystem Therapeutic 4 (MET4) consisting of 30 bacterial species	- Initially 20 capsules over 2 d (2–10 × 10^10^ CFU), followed by 3 capsules daily (6–30 × 10^9^ CFU) - Taken for ≤1 y or until toxicity, disease progression, or treatment discontinuation.	No probiotic treatment	NS Δ in GIAEs between groups: • Manageable diarrhea and constipation • No grade ≥3 GIAEs ORR ↑ in probiotic group: • ORR: 35% (probiotic) vs. 14% (controls) • CR: 1 vs. 1 • PR: 7 vs. 1 • SD: 6 vs. 3 • Clinical benefit rate: 53% vs. 20%	RECIST v1.1 criteria and iRECIST. CTCAE v5.0.	RCT	Moderate
Biological therapy)											
Cong et al. [48], 2022	China	Non–small cell lung cancer	Bevacizumab (VEGF-TKI) (15 mg/kg every 3 wk) + platinum-based chemotherapy (paclitaxel and carboplatin) until disease progression or intolerance.	*N:* 21 (9, 12) 29% F Mean 65 y	Probiotic: *C*. *butyricum*	- 6 pills/d (420 mg/pill), viable count ≥ 6.3 × 10^6^ CFU - Administered alongside chemotherapy cycles	Placebo: similar packaging, only starch and glucose—the medium in which the *C. butyricum* strains were removed.	GIAEs ↓ in probiotic group vs. control: • Diarrhea: 0% (probiotic) vs. 41.7% (control) • Nausea: 11.1% vs. 25% • Vomiting: 0% vs. 16.7% • Constipation: 0% vs. 8.3% NS Δ in PFS and OS: • PFS: 174d (probiotic) vs. 187d; *P* = 0.799 • OS: 415d vs. 421d; *P* = 0.941	RECIST v1.1.	RCT	Moderate
Dizman et al. [54], 2021	USA	Metastatic renal cell carcinoma	VEGF-TKIs: cabozantinib, sunitinib, lenvatinib/everolimus, and axitinib.	*N:* 20 (10, 10) 25% F 32–81 y	Probiotic: Probiotic yogurt containing *B*. *animalis*	- 2 servings (4 oz. each) - Taken daily for 3 mo	No probiotic treatment	NS Δ in diarrhea between groups: • Grade 1–2 diarrhea in both groups: 40% • No grade 3/4 events ORR ↑ in probiotic group: • PR: 20% (probiotic) vs. 10% (control) • Stable disease: 50% vs. 70% • Progressive disease: 10% vs. 20% • Median PFS: 6.2 vs. 13.8 mo	CTCAE v4.0.	RCT	Moderate
Ianiro et al. [61], 2020	Italy	Metastatic renal cell carcinoma	TKIs: sunitinib and pazopanib	*N:* 20 (10, 10) 25% F Mean 65 y	FMT	- ∼57 g stool from 1 donor, diluted in saline, delivered via colonoscopy - Bowel cleansing with 4 L of macrogol day before colonoscopy - Single treatment with follow-up at weeks 1, 2, 4, and 8	Placebo: FMT via colonoscopy of 250-mL water	Diarrhea incidence ↓ after FMT: • Diarrhea at 4 wk: 30% (FMT) vs. 100% (control); *P* = 0.003 • Diarrhea at 1 wk: 0% vs. 30%; *P* = 0.02	CTCAE v4.0	RCT, bb	Moderate

Abbreviations: b, single-blind; bb, double-blind; CR, complete response; CTCAE, Common Terminology Criteria for Adverse Events; EORTC QLQ-C30, European Organization for Research and Treatment of Cancer quality-of-life Questionnaire C30; F, female; GIAE, gastrointestinal adverse event; HR, hazard ratio; IV, intravascular; IM, intramuscular; *N*, sample size (total number of participants); NS, nonsignificant; NSΔ, no significant difference/change between groups; OR, odds ratio; ORR, objective response rate; PR, partial response; QoL, quality of life; RECIST, Response Evaluation Criteria in Solid Tumors; RR, relative risk; TKI, tyrosine kinase inhibitor; VEGF, Vascular Endothelial Growth Factor; Δ, change; ↓, decrease/lower; ↑, increase/higher.

**TABLE 2:** Characteristics of prebiotic studies

Study	Country	Malignancy	Cancer treatment regimen	Participants	Intervention	Dose and length of intervention	Comparator	GIAE and response outcomes	Outcome reporting method	Study design	Study quality rating
Radiotherapy (RT))											
Forslund et al. [84], 2020	Sweden	Prostate cancer	Intensity- modulated RT, volumetric modulated arc therapy or rapid arc technique, to prostate, seminal vesicles, and pelvic lymph nodes + prostate boost	*N:* 180 (92, 88) 100% M Mean: 67.2 ± 5.4 y	Prebiotic: soluble fiber diet	- 3 sessions with a dietitian (baseline, 4 wk, and 8 wk) - Advised to replace insoluble fiber and lactose with soluble fiber and low-lactose foods for 26 mo - Dietary pamphlet provided at baseline + mailed reminders	Advised to maintain habitual diet, no routine dietary counseling but dietitian consultation available if needed	NS Δ for GIAE scores: • Diarrhea: 35 (prebiotic) vs. 31 (control) • Constipation: 10 vs. 12 Blood in stools and flatulence ↓ in prebiotic group: • Blood in stools: *P* = 0.047 • Flatulence: *P* = 0.014	EORTC QLQ-C30	RCT	Moderate
Garcia-Peris et al. [58], 2016	Spain	Gynecologic cancer	Postoperative radiation: 1.8 Gy daily, 5 d a week for 29 d using linear accelerator (15 MV), followed by brachytherapy 1 wk later	*N:* 38 (20, 18) 100% F 36–77 y	Prebiotic: inulin (50%) Fructo-oligosaccharide (FOS) (50%) (+ dietary counseling)	- 6 g powder, 2×/d (dissolved in 200-mL water) - From 1 wk before RT to 3 wk after	Placebo: maltodextrin (+dietary counseling)	Watery stools ↓ in prebiotic group (*P* = 0.08) vs. placebo • NS Δ time to diarrhea onset or watery stool presence • NS Δ in symptom scores for diarrhea, constipation or nausea/vomiting • NS Δ overall health scale (QoL)	CTCAE, Bristol Stool Scale, and EORTC-QLQ-C30	RCT, bb	Strong
Murphy et al. [91], 2000	Canada	Prostate and gynecologic cancer	RT to the pelvis of ≥4000 cGy in 20 fractions were recruited from the 2 site loca	*N:* 60 (30, 30) 15% F Mean: 62.9 (46–88) y	Prebiotic: metamucil (psyllium husk)	- Researcher advised diet education: low-fiber diet, limited fat, caffeine, and alcohol - Intervention group instructed on metamucil use	No metamucil treatment	Diarrhea frequency and severity ↓ in prebiotic group: • Diarrhea incidence: 60% (prebiotic) vs. 83% (control); *P* =0.049 • Severe diarrhea incidence: 37% vs. 57%; *P* = 0.03	Self-reported by patient and physician	RCT	Moderate
Pettersson et al. [87], 2012	Sweden	Prostate cancer	External beam RT (2 Gy/d, total 50 Gy) + high-dose-rate brachytherapy (2 × 10 Gy) (*n* = 80) or proton therapy (perineal boost 4 × 5 Gy) (*n* =50) over 7 wk	*N:* 130 (64, 66) 100% M Median: 66 (50–77 y)	Prebiotic: soluble fiber diet	- Standardized dietary guidance via face-to-face sessions, phone calls, and brochures - Avoid insoluble fiber/lactose; consume soluble fiber/low-lactose foods - From RT initiation to 24 mo post-RT	No dietary counseling, advised to continue with normal diet	NS Δ in GIAE symptoms: • Diarrhea: 13% (prebiotic) vs. 19% (control) • Constipation: 12% vs. 8% Bloated abdomen and stool leakage ↓ in prebiotic group: • Bloated abdomen: 33% vs. 43% • Stool leakage: 9% vs. 20%	EORTC QLQ-C30	RCT	Moderate
Rosli et al. [89], 2021	Malaysia	Endometrium, cervix, colon, rectum, and prostate cancers	External beam RT: ≥40 Gy (1.8 - 2.2 gray/fraction)	*N:* 30 (14, 16) 73.3% F Mean: 56.0 ± 10.9 y	Prebiotic: partially hydrolyzed guar gum (PHGG)	- 10 g PHGG or placebo in 100-mL water, 2×/d (before breakfast and dinner) - Taken 14 d before RT + 14 d during RT	Placebo: maltodextrin	Mean diarrhea frequency ↓ in prebiotic group at end of RT (*P* < 0.05) (2 wk postsupplementation): • 54.5% (prebiotic) vs. 58.3% (control) NS Δ in global health score	CTCAE, Bristol stool criteria, and EORTC-QLQ-C30	RCT, bb	Strong
Chemoradiotherapy)											
Flores-Cisneros et al. [83], 2025	Mexico	Cervical cancer	Pelvic RT: 50 Gy in 25 fractions. Chemotherapy (cisplatin or gemcitabine) ≥3 cycles/wk; 5 high-dose brachytherapy sessions	*N:* 134 (65, 69) 100% F Mean: 48.5 ± 13.8 y	Prebiotic: high-fiber diet	- Individualized diet (30–40 kcal/kg/d) - 25 g fiber (20 g soluble, 5 g insoluble) and 5 g lactose distributed over 5 meals - Macronutrient composition: 55%–60% carbs, 0.8–1.3 g/kg protein, 20% fat - Support: Verbal instructions + nutritional handbook - No information on duration	Standard diet: no whole grains, raw fruits/vegetables, dairy, coffee, or carbonated drinks allowed	Constipation ↓ in prebiotic group: • Third cycle: 30.7% (prebiotic) vs. 37.6% (control) • Adjusted HR (third cycle and end of treatment): 0.46 (95% CI: 0.28, 0.76; *P* < 0.01) • Fiber intake >18.7 g/1000 kcal led to ↓ constipation (*P* < 0.05) NS Δ in other GIAEs (third cycle): • Diarrhea: 50.77% vs. 50.72% • Diarrhea ≥ grade 3: 0% vs. 2.9% • Nausea: 66.2% vs. 66.7% • Vomiting: 18.5% vs. 18.8%	CTCAE v4.0	RCT	Moderate
Sasidharan et al. [76], 2019	India	Carcinoma cervix	RT: 50 Gy in 25 fractions over 5 wk, using cobalt-60 or 6/15 MV linear accelerator. Weekly cisplatin 40 mg/m^2^ (3–4 cycles) with brachytherapy boost	*N:* 100 (50, 48) 100% F 25–68 y	Prebiotic: Nondigestible, amylase resistant starch [high-amylose maize starch (HAMS)]	- 30 g of HAMS 2×/d during RT, mixed with 150-mL milk or water - From start of RT until end of RT (6 wk)	Placebo: 30 g regular digestible maize starch (25% amylose)	Diarrhea ↓ in probiotic group: • At week 4: 14% (prebiotic) vs. 14.6% (control)	CTCAE v3.0 and Radiation Therapy Oncology Group toxicity scales	RCT, bb	Moderate
Wedlake et al. [80], 2017	England	Gynecologic or lower gastrointestinal cancer including colorectal, anal, and cervical cancers	Pelvic RT, ≥ 45 Gy, 1.8 Gy daily, 5 times weekly for 5–7 wk. Patients with gynecologic cancer received brachytherapy. Concomitant chemotherapy: oral capecitabine and mitomycin C for colorectal/anal cancers, weekly cisplatin for cervical cancer	*N:* 128 (45, 83) 75% F 26–91 y	Prebiotic: High-fiber (HF) diet with 2 groups: 1. HF [≥18 g nonstarch polysaccharide (NSP)/d]. 2. Low-fiber (LF) (≤10 g NSP/d)	- Patients had enrollment and exit interviews with a dietitian, plus on-treatment interviews during RT - Daily fiber target set, with counseling on achieving it through diet (no fiber supplements provided or recommended) - Counseling focused on food choices, emphasizing fiber-rich options, and adjusting them to meet the target	Maintain normal diet, no adjustments to fiber intake, no educational materials provided	NS Δ in stool frequency or form (*P* = 0.934) • End-of-RT stool form 6/7 (d): 3.0 (HF) vs. 3.0 (LF) vs. 3.0 (control); *P* = 0.934 • End-of-RT IBDQ-B scores: HF: 58.0, LF: 56.0, control: 53.3 (*P* = 0.104). • Significant Δ in IBDQ-B between groups from baseline to end of RT (*P* = 0.014).	Bristol stool scale and Inflammatory Bowel Disease Questionnaire-Bowel Subset (IBDQ-B)	RCT	Weak
Chemotherapy)											
Becerril-Alarcón et al. [46], 2019	Mexico	Breast cancer	Cyclophosphamide and doxorubicin (600 mg/m^2^/60 mg/m^2^) by infusion	*N:* 38 (19, 19) 100% F Mean: 51.5 ± 9.3 y	Prebiotic: inulin	- 5 g agave inulin dissolved in 240-mL water, consumed at breakfast - Taken starting on the first day of chemotherapy for 21 d	Placebo: 15 g of maltodextrin	NS Δ in GIAE incidence: • Nausea/vomiting: 26% (prebiotic) vs. 47% (control) • Constipation: 21% vs. 47% • *P* = NS	EORTC-QLQ-C30	RCT, bb	Strong
D’Amico et al. [49], 2022	Italy	Acute lymphoblastic leukemia, acute myeloid leukemia and non-Hodgkin lymphoma	First-line induction chemotherapy	*N:* 34 (14, 20) 1.49–18.35 y	Prebiotic: Bovine lactoferrin	- 200 mg/d of bovine lactoferrin, taken daily for 2 mo - Started with induction chemotherapy	Placebo	Febrile neutropenia ↓ in prebiotic group: • 57.1% (prebiotic) vs. 90% (control); *P* = 0.04 No adverse effects reported in any patient	NA	RCT, bb	Strong
Rathe et al. [73], 2020	Denmark	Acute lymphoblastic leukemia	Chemotherapy	*N:* 62 (30, 32) 48% F 1–15 y	Prebiotic: bovine colostrum powder	- Colostrum: 0.5–1 g/kg/d - Taken daily from the first day of chemotherapy (or first day of consent) until day 29 or end of induction therapy - Given as a single dose or divided into 2 or 3 daily doses based on child’s preference	Placebo: prepared from whole-milk powder	Diarrhea ↓ in prebiotic group (*P* = 0.06): • Grade 0: 55.2% (prebiotic) vs. 71.0% (control) • Grade 1: 37.9% vs. 12.9% • Grade 2: 0% vs. 9.7% • Grade 3: 6.9% vs. 6.5%	CTCAE v4.0	RCT, bb	Strong
Valadares et al. [18], 2013	Brazil	Breast cancer	Chemotherapy: CMF (cyclophosphamide, methotrexate, 5-FU) or FAC (5-FU, doxorubicin, cyclophosphamide), 21-d cycles.	*N:* 46 (23, 23) 100% F Mean: 51.1 y	Prebiotic: *Agaricus sylvaticus* (sun mushroom)	- 2.1 g fungal supplement daily, split into 2 doses - Six tablets per day (3 morning, 3 afternoon, between meals) for 6 mo	Placebo	GIAEs ↓ in prebiotic group: • Diarrhea: 4% (prebiotic) vs. 39% (control) • Nausea: 26% vs. 83% • Vomiting: 17% vs. 70% • Constipation: 13% vs. 30%	Self-reported	RCT, bb	Strong

b, single-blind; bb, double-blind; CR, complete response; CTCAE, National Cancer Institute Common Terminology Criteria for Adverse Events; EORTC QLQ-C30, European Organization for Research and Treatment of Cancer quality-of-life Questionnaire C30; F, female; GIAE, gastrointestinal adverse event; IBDQ-B, Inflammatory Bowel Disease Questionnaire-Bowel Subset; HR, hazard ratio; *N*, sample size (total number of participants); NS, nonsignificant; NS Δ, no significant difference/change between groups; OR, odds ratio; ORR, objective response rate; PR, partial response; QoL, quality of life; RECIST, Response Evaluation Criteria in Solid Tumors; RR, relative risk; Δ, change; ↓, decrease/lower; ↑, increase/higher.

**TABLE 3:** Characteristics of synbiotic studies

Study	Country	Malignancy	Cancer treatment regimen	Participants	Intervention	Dose and length of intervention	Comparator	GIAE and response outcomes	Outcome reporting method	Study design	Study quality rating
Radiotherapy (RT))											
Nascimento et al. [71], 2020	Brazil	Prostate cancer	Total dose: 66–76 Gy, daily dose: 2 Gy, 6–8 wk Monday to Friday with weekend interval	*N:* 20 (10, 10) 0% F Mean: 67 y	Synbiotic: Inulin Guar gum *L*. *reuteri*	- 5 g sachets of synbiotic powder containing 4.3 g inulin + hydrolyzed guar gum - >10^8^ CFU L. reuteri - 1 sachet daily in water a week before RT - 2 sachets daily after RT sessions begin	Placebo: maltodextrin (5 g)	Proctitis symptoms and QoL scores ↓ in synbiotic group: • Week 5 scores: 22 (21–34) (synbiotic) vs. 26 (21–47) (placebo); *P* =0.035 • Week 6 scores: 21 (22–33) vs. 29 (21–35); *P* =0.017	EORTC QLQ Proctitis module for RT-treated patient questionnaire	RCT, bb	Strong
Chemoradiotherapy)											
De Loera Rodríguez et al. [50], 2018	Mexico	Cervical cancer	Treatment with chemotherapy and radiotherapy	*N:* 70 (35, 35) 100% F Mean: 50 y	Synbiotic: *L. acidophilus* NCFM biogel, *B*. *lactis* Bi-07 biogel, and blue agave inulin	- Synbiotic contains 1 × 10^7^ CFU/g *L. acidophilus* NCFM and 1 × 10^7^ CFU/g B. lactis - 20 g gels - 1 gel 3×/d (30 min before breakfast, lunch, dinner) for 7 wk	Placebo gel	Vomiting ↓ in synbiotic group during treatment (*P* = 0.025): • Grade 1 vomiting: 94.3% (synbiotic) vs. 91.4% (placebo) • Grade 2 vomiting: 5.7% vs. 11.4% NS Δ for other GIAEs: • Diarrhea: 14.3% (synbiotic) vs. 20.0% (placebo) • Constipation: 8.6% vs. 11.4% • Grade 1 Nausea: 88.6% vs. 88.6% • Grade 2 Nausea: 11.4% vs. 11.4% • Stool consistency: NS Δ after 7 wk.	Bristol Stool Scale, National Institute of Cancerology of the United States nausea, and vomiting scale	RCT, bb	Strong
Fukaya et al. [57], 2021	Japan	Esophageal cancer	Chemotherapy: 2 cycles of cisplatin (80 mg/m^2^) on days 1 and 22, 5-FU (800 mg/m^2^) on days 1–5 and 22–26. RT conducted 4–5 wk postchemotherapy in some patients.	*N:* 42 (22, 20) 12% F 44–77 y	Synbiotic: *L*. *paracasei* strain Shirota, *B. breve*, and galacto-oligosaccharides	- 80-mL Yakult 400 (≥4 × 10^10^ *L. paracasei Shirota*) - 100 mL MILMIL-S (≥1 × 10^10^ *B. breve* Yakult) - 15 g Oligomate S-HP (≥4.95 g galacto-oligosaccharides) - Daily from 7 d before neoadjuvant chemotherapy to 1 d before surgery	No synbiotics treatment	≥Grade 3 GIAEs ↓ in synbiotic group (*P* = 0.022) NS Δ for all grades: • Diarrhea (any): 20% (synbiotic) vs. 32% (control) • ≥Grade 3 diarrhea: 0% vs. 5% • Nausea (any): 40% vs. 64% • ≥Grade 3 nausea: 0% vs. 9% • Vomiting: 5% vs. 0% (no ≥grade 3) • Constipation: 30% vs. 36% (no ≥grade 3) ORR ↑ in synbiotic group: • Partial response: 60% (synbiotic) vs. 56% (control) • No complete responses	CTCAE v5.0. RECIST v1.1	RCT	Moderate
Scartoni et al. [77], 2015	Italy	Endometrial, cervical, anal, colorectal, and prostate cancer	Pelvic RT with 15 MV and 18 MV photons, total dose 63.3 Gy. Concomitant chemotherapy in 5 cases: capecitabine for gastrointestinal cancer, cisplatin for gynecologic cancer	*N:* 40 (40, 0) 23% F	Synbiotic: *L. casei*, *L. acidophilus*, galacto-oligosaccharides, zinc, vitamin B-1, vitamin B-2, vitamin B-6 and nicotinamide (Dixentil)	- 10 --mL vial of Dixentil daily from the first day of RT until the end - Each vial contains 500-mg galacto-oligosaccharides, 10-mg *L. casei*, 10-mg *L. acidophilus*, 10-mg zinc, 1 mg each of vitamins B-1, B-2,and B-6, and 10-mg nicotinamide - Two vials on the first day, then 1 vial daily thereafter	No control group	Diarrhea frequency low: • Grade 1: 35% • Grade 2: 7.5% • Grade 3/4: 0% • No control group	CTCAE v4.0	Non-RCT	Moderate
Chemotherapy)											
Eghbali et al. [56], 2023	Iran	Acute lymphoblastic leukemia	Maintenance chemotherapy	*N:* 106 (54, 52) 42% F 5–15 y	Synbiotic: Lactocare: *L*. *casei*, *L. acidophilus*, *L. rhamnosus*, *L. bulgaricus*, *B. breve*, *B*. *longum*, *S. thermophiles*, and prebiotic fructo-oligosaccharides	- 5 × 10^9^ CFU of LactoCare taken orally 2×/d for 7 d - Starting on first day of chemotherapy	Placebo: starch capsules	GIAEs ↓ in synbiotic group: • Diarrhea: 0% (synbiotic) vs. 5.8% (control), OR = 1.45, 95% CI: 1.17–4.01; *P* = 0.048 • Nausea: 1.9% vs. 11.5% • Vomiting: 0% vs. 11.5% • Constipation: 0% vs. 17.3%	Bristol Stool Scale. Self-reported: recorded by patient’s parents in a notebook according to a guidance sheet.	RCT, bb	Strong
Ghaffari et al. [59], 2024 (Translated from Arabic)	Iran	Nonhematologic neoplasms including neuroblastoma, central nervous system tumor, AML, Ewing sarcoma, myosarcoma, and lymphoma	Chemotherapy for 14 d	*N:* 88 (46, 42) 49% F 5–15 y	Synbiotic: Lactocare: *L*. *rhamnosus*, *L. casei*, *L. acidophilus*, *L. bulgaricus*, *L. plantarum*, *L. gasseri*, *L. helveticus*, *B. lactic*, *B. breve*, *B. longum*, *B. bifidum*, *S. thermophiles*, and fructo-oligosaccharides	- 5 × 10^9^ CFU of LactoCare taken orally 2×/d for 14 d - Starting on first day of chemotherapy	Placebo	Control group 1.37× more likely to develop diarrhea (*P* = 0.041) • Fewer days of diarrhea and constipation in synbiotic group (*P* < 0.05) NS Δ in nausea/vomiting severity	Self-reported: parents reported incidence GIAEs	RCT, bb	Strong
Khazaei et al. [64], 2023	Iran	Breast cancer	Adriamycin and cyclophosphamide every 4 wk	*N:* 67 (34, 33) 100% F Mean: 52.29 ± 11.94 y	Synbiotic: Lactocare: *L*. *rhamnosus*, *L. casei*, *L. acidophilus*, *L. bulgaricus*, *B*. *breve*, *B. longum*, *L. helveticus*, *L. lactis*, *L. paraplantarum*, *B. bifidum*, *S. thermophilus*, *L. gasseri*, and fructo-oligosaccharides as a prebiotic	- Each 450-mg capsule contains 12 probiotic strains (1 × 10^9^ CFU) and 21 g of fructo-oligosaccharides - One capsule taken 2×/d after main meals for 8 wk (112 capsules)	Placebo capsule: starch	GIAEs ↓ in synbiotic group: • Diarrhea at week 8: 2.9% (synbiotic) vs. 87.8% (control); *P* = 0.005 • Constipation: 2.9% vs. 36.4% • Nausea/vomiting score: 17.17 (probiotic) vs. 22.06 (control); *P* = 0.394	Bristol Stool Scale and Rhodes index of nausea and vomiting—form2 index (↑ score = ↑ nausea/vomiting)	RCT, bb	Strong
Mohebian et al. [19], 2023	Iran	Colorectal cancer	5-FU chemotherapy	*N:* 66 (41, 25) 68% F Mean: 56.5 ± 6.59 y	Synbiotic: *L. casei*, *L. acidophilus*, *L. rhamnosus*, *L. bulgaricus*, *B. breve*, *B. longum*, *S. thermophiles*, and fructo-oligosaccharides and yogurt	- Synbiotic capsules: 500 mg - Treatment group: 150 g yogurt + 1 capsule 2×/d (before breakfast and dinner) for 1 wk - 2 control groups: *1*) Placebo group: 150 g yogurt 2×/d (before breakfast and dinner) for 1 wk *2*) Control group: no intervention	2 controls: *1*) Placebo *2*) No treatment	Diarrhea severity ↓ in synbiotic vs. control (*P* < 0.05) • Day 3 severity: 0.74 (synbiotic) vs. 0.91 (placebo) vs. 1.76 (control); *P* = 0.001 • ↑ Stool consistency on days 4–5 in synbiotic vs. control	Self-reported table using CTCAE grades and patient-reported form	RCT	Moderate
Motoori et al. [69], 2017	Japan	Esophageal cancer	Neoadjuvant DCF chemotherapy: docetaxel (70 mg/m^2^) + cisplatin (70 mg/m^2^) IV on day 1, 5-FU (700 mg/m^2^) continuous infusion on days 1–5. two cycles, 3 wk apart	*N:* 61 (30, 31) 8% F Mean: 64 y	Synbiotic: *B. breve* strain Yakult, *L. casei* strain Shirota, and galacto-oligosaccharides (Yakult BL Seichoyaku)	- 3 g of synbiotic (or Biofermin) daily - 1 × 10^8^ CFU of each bacterial strain in synbiotic - 15 g of prebiotic daily - Treatment started 2 d before chemotherapy and continued for 6 wk	Probiotic: Biofermin containing 1 × 10^9^*S*. *faecalis*	Diarrhea ↓ in synbiotic vs. probiotic group (*P* = 0.035) • Grade 1: 40% (synbiotic) vs. 38.7% (probiotic) • Grade 2: 26.7% vs. 25.8% • Grade 3: 10% vs. 25.8% • Grade 4: 0% vs. 3.2%	CTCAE v4.0	RCT	Moderate
Motoori et al. [70], 2022	Japan	Esophageal cancer	Neoadjuvant DCF chemotherapy: Docetaxel (70 mg/m^2^) + Cisplatin (70 mg/m^2^) IV on day 1, 5-FU (700 mg/m^2^) continuous infusion on days 1–5. Two cycles, 3 wk apart.	*N:* 81 (40, 41) 74% F 37–79 y	Synbiotics and enteral nutrition: *B. breve* strain Yakult and *L. paracasei* strain Shirota, galacto-oligosaccharides (Yakult BL Seichoyaku), ω-3 fatty acid–rich enteral nutrition	- 1 × 10^8^ of each living strain. - 3-g probiotic and 15-mL prebiotic daily, starting 3 d before chemotherapy until its end. - 600-mL enteral nutrition daily, from 3 d before chemotherapy until day 12.	Antibiotic: 500 mg/d of levofloxacin orally, days 5–15 of each chemotherapy course	Diarrhea ↓ in synbiotic vs. antibiotic group: • Grade 0–1: 67.5% (synbiotic) vs. 43.9%(antibiotic) • Grade 2–4: 32.5% vs. 56.1%; *P* = 0.003	CTCAE v4.0	RCT	Moderate
Wei et al. [17], 2024	China	Lung cancer (including non–small cell lung cancer and small cell lung cancer)	Platinum-based doublet chemotherapy: cisplatin/carboplatin + paclitaxel, docetaxel, gemcitabine, vinorelbine, pemetrexed, or etoposide.	*N:* 91 (42, 49) 31% F Mean: 59.5 ± 8.09 y	Synbiotic: *B*. *lactis* Bi-07, *L. acidophilus* NCFM, *L. rhamnosus* HN001, *B. lactis* HN019, and oligofructose (added at >93.69%)	-One sachet (2 g) dissolved in water, 2×/d -Started with first chemotherapy cycle until the third cycle	Placebo: maltodextrin	Diarrhea ↓ in synbiotic vs. control group: • Any diarrhea: 7.1% (synbiotic) vs. 42.9% (control); *P* < 0.001 • Grade 1: 7.1% vs. 26.5% • Grade 2: 0% vs. 14.3% • Grade 3: 0% vs. 2.0% Nausea/vomiting ↓ in synbiotic vs. control group: • 16.7% vs. 71.4%; *P* < 0.001 • Grade 3: 0% vs. 4.1% Constipation ↓ in synbiotic vs. control group: • 16.7% vs. 63.3%; *P* < 0.001	CTCAE v4.0. and EORTC QLQ C30	RCT, bb	Strong

Abbreviations: b, single-blind; bb, double-blind; CR, complete response; CTCAE, Common Terminology Criteria for Adverse Events; EORTC QLQ-C30, European Organization for Research and Treatment of Cancer quality-of-life Questionnaire C30; F, female; GIAE, gastrointestinal adverse event; HR, hazard ratio; IV, intravascular; IM, intramuscular; *N*, sample size (total number of participants); NS, nonsignificant; NSΔ, no significant difference/change between groups; OR, odds ratio; ORR, objective response rate; PR, partial response; QoL, quality of life; RECIST, Response Evaluation Criteria in Solid Tumors; RR, relative risk; TKI, tyrosine kinase inhibitor; VEGF, Vascular Endothelial Growth Factor; Δ, change; ↓, decrease/lower; ↑, increase/higher.

**TABLE 4:** Characteristics of FMT studies

Study	Country	Malignancy	Cancer treatment regimen	Participants	Intervention	Dose and length of intervention	Comparator	GIAE and response outcomes	Outcome reporting method	Study design	Study quality rating
Immunotherapy											
Baruch et al. [24], 2021	Israel	Melanoma	Nivolumab (anti–PD-1). All patients were primary refractory (no prior response to anti–PD-1).	*N:* 10 (10, 0) 30% F 49–68 y	FMT	FMT: colonoscopy + oral capsules after antibiotic preconditioning (vancomycin + neomycin) from anti–PD-1 responders - Duration: 6 FMT cycles with nivolumab every 14 d for 90 d - Continuation: responders stayed on anti–PD-1 until progression	No direct comparator; results compared with historical anti–PD-1 response rates.	GIAEs: • Grade 1 bloating attributed to FMT Response: • ORR: 30% (CR: 10%, PR: 20%) • Tumor pseudoprogression: 20% • All responders surpassed 6-mo progression-free survival.	Immune RECIST (iRECIST) criteria. No information for GIAE evaluation	Non-RCT	Moderate
Davar et al. [25], 2021	USA	Melanoma	Pembrolizumab (anti–PD-200 mg every 3 wk, with scans every 9–12 wk). ≤35 cycles or until progression/toxicity. All patients were primary refractory (no response to anti–PD-1 alone or with CTLA-4/investigational agents)	*N:* 16 (16, 0) 25% F 35–85 y	FMT	- FMT: single colonoscopic dose from long-term anti–PD-1 responders	No control comparator: historical data of anti–PD-1 therapy alone	GIAEs: • Nausea and vomiting: 13.3% • Diarrhea: 12.5% Response: • ORR: 20% • Disease control rate: 40% (3 with durable stable disease >12 mo) • Median PFS: 3 mo (14 mo in those with disease control) • Median OS: 7 mo (14 mo in those with disease control).	RECIST v1.1. No information for GIAE evaluation	Non-RCT	Moderate
Routy et al. [74], 2023	USA	Melanoma	Anti–PD-1 immunotherapy (nivolumab or pembrolizumab) every 3–4 wk for ≤12 mo. All patients previously untreated.	*N:* 20 (20, 0) 40% F 48–90 y	FMT	- Single FMT via oral capsules from healthy donors, 1 wk before anti–PD-1 therapy.	No control; outcomes compared with historical anti–PD-1 data.	GIAE: • Diarrhea: 30% • Nausea: 5% • No grade 3 GIAEs Response: • ORR: 65% • CR: 20% • PR: 45% • Clinical Benefit Rate: 75% (CR + PR + stable disease ≥6 mo). • PFS: Median not reached after 20.7 mo; 80% patients alive at cutoff.	RECIST v1.1 criteria	Non-RCT	Moderate
Biological therapy											
Ianiro et al. [61], 2020	Italy	Metastatic renal cell carcinoma	TKIs: sunitinib and pazopanib	*N:* 20 (10, 10) 25% F Mean: 65 y	FMT	- ∼57 g stool from 1 donor, diluted in saline, delivered via colonoscopy - Bowel cleansing with 4 L of macrogol day before colonoscopy - Single treatment with follow-up at weeks 1, 2, 4, and 8	Placebo: FMT via colonoscopy of 250-mL water	Diarrhea incidence ↓ after FMT: • Diarrhea at 4 wk: 30% (FMT) vs. 100% (control); *P* = 0.003 • Diarrhea at 1 wk: 0% vs. 30%; *P* = 0.02	CTCAE v4.0	RCT, bb	Moderate

Abbreviations: b, single-blind; bb, double-blind; CR, complete response; CTCAE, Common Terminology Criteria for Adverse Events; EORTC QLQ-C30, European Organization for Research and Treatment of Cancer quality-of-life Questionnaire C30; F, female; FMT, fecal microbiota transplantation; GIAE, gastrointestinal adverse event; HR, hazard ratio; *N*, sample size (total number of participants); NS, nonsignificant; NSΔ, no significant difference/change between groups; OR, odds ratio; ORR, objective response rate; OS, overall survival; PFS, progression-free survival; PR, partial response; QoL, quality of life; RECIST, Response Evaluation Criteria in Solid Tumors; RR, relative risk; TKI, tyrosine kinase inhibitor; VEGF, Vascular Endothelial Growth Factor; Δ, change; ↓, decrease/lower; ↑, increase/higher.

RCTs dominated the review, with only 2 nonrandomized clinical trials [51,55], and 4 single-arm studies (pre-post) [24,25,74,77]. Of the 50 RCTs, 27 were double-blind [[16], [17], [18],[45], [46], [47],^49^,^50^,^52^,^53^,^56^,[58], [59], [60], [61], [62], [63], [64], [65],^67^,^68^,^71^,^73^,^76^,^81^,^85^,^89^, ^3^[16–18,45–47,49,50,52,53,56,58–65,67,68,71,73,76,81,85,89], 3 single-blind [15,78,88], and 20 open-label [19,27,28,48,54,57,66,69,70,72,75,79,80,[82], [83], [84],^86^,^87^,^90^,^91^] studies.

Control groups and conditions varied in rigor among the included RCTs. Eighteen probiotic studies and 7 synbiotic studies used placebo controls [[15], [16], [17],^19^,^45^,^47^,^48^,^52^,^53^,^55^,^59^,^60^,^62^,^63^,[65], [66], [67], [68],^71^,^78^,^81^,^85^]. Some studies used no active control condition, comparing no supplementation with probiotic [27,28,51,54,72,75,79,82,86,88], synbiotic [57], or prebiotic supplementation [91]. Among synbiotic studies, one compared synbiotics to probiotics [69], and another used a prophylactic antibiotic group as control [70].

The biggest variation in type of controls used were observed in prebiotic studies, as these ranged from digestible maize starch as a placebo for resistant starch [76] to habitual diet as the control for high-fiber diet interventions [84,87]. One study compared 3 different fiber intake interventions [low <10 g/d nonstarch polysaccharides (NSP), habitual and high intake >18 g/d NSP] [80], whereas only 1 study provided a standardized restricted diet (excluding whole grains, raw fruits/vegetables, dairy, and stimulants) as the control condition to a high-fiber diet intervention [83].

### Intervention characteristics

#### Probiotics

Probiotics were the most studied intervention, used in 29 trials during radiotherapy, chemoradiotherapy, or chemotherapy, particularly for pelvic (e.g., cervical, prostate, and gynecologic) [45,47,[51], [52], [53],^60^,^65^,^66^,^75^,^90^] and colorectal [15,67,68,72,79,90] cancers. The most frequently used organisms were *Lactobacillus acidophilus* and *Bifidobacterium longum*, primarily within multistrain formulations (2–10 strains) also incorporating *Lactobacillus casei, Lactobacillus rhamnosus, Lactiplantibacillus plantarum, Streptococcus thermophilus*, and *Enterococcus faecalis*. Standalone strains included *Clostridium butyricum* [16,27,48,79,82], *L. rhamnosus GG* [88], and *L. acidophilus* [75]. Novel combinations were also tested, such as *Bacteroides fragilis BF839* [81] and the 30-species Microbial Ecosystem Therapeutic MET4 [28]. Capsules/tablets were the predominant delivery vehicles, but sachets, fermented yogurt [54,60], and freeze-dried powders [78] were also used.

Of the 29 trials using probiotic supplementation, 19 studies reported the dosage used [15,27,28,45,47,48,52,53,[65], [66], [67], [68],^72^,^75^,^78^,^79^,^81^,^82^,^88^], which ranged from 1.65 × 10^6^ to 1.35 × 10^12^ CFU/d, with a mean of 8.8 × 10^10^ and median of 8.1 × 10^9^ CFU/d. Higher doses were generally used in multistrain combinations. Most trials administered probiotics 2 or 3 times per day: 12 studies used thrice-daily dosing [15,16,28,51,52,55,60,65,67,68,78,79], whereas 14 studies opted for twice-daily [27,45,47,53,54,62,63,66,72,81,82,85,86,88,90] and only 1 used once-daily dosing [75].

Initiation relative to cancer treatment varied. Seven studies began probiotic administration on the first day of therapy [[51], [52], [53],^65^,^79^,^81^,^90^], whereas 8 initiated supplementation 1–14 d prior [16,45,47,55,60,66,75,78]. Most interventions continued throughout the treatment period, with some extending beyond: durations ranged from 1 wk [88] to as long as 1 y [28].

#### Prebiotics

Of the 12 prebiotic studies, 8 involved patients with pelvic and colorectal cancer receiving radiotherapy or chemoradiotherapy [58,76,80,83,84,87,89,91], and 4 targeted chemotherapy patients with breast cancer [18,46] or pediatric acute lymphoblastic leukemia (ALL) [49,73]. The studies used a diverse range of prebiotic types, including inulin/fructo-oligosaccharide mixtures [58], psyllium husk [91], partially hydrolyzed guar gum [89], high-amylose maize starch [76], agave inulin [46], and fungal extract from *Agaricus sylvaticus* [18]. Additionally, 4 studies used dietary strategies to increase soluble fiber intake [80,83,84,87], and 2 used bioactive protein-based interventions: bovine lactoferrin [49] and bovine colostrum [73].

The daily dose of prebiotic supplementation used varied substantially across studies. These range from 60 g/d maize starch [76, 20[76], 20 g/d guar gum [89, 12[89], 12 g/d inulin/fructo-oligosaccharides [58, 5[58], 5 g/d agave inulin [46], and 2.1 g/d *A*. *sylvaticus* [18]. High-fiber diet studies specified intake targets rather than supplements: Flores-Cisneros et al. [83] prescribed 25-g total fiber per day (20 g soluble, 5 g insoluble) plus 5-g lactose, distributed across 5 meals; Wedlake et al. [80] set group targets of ≥18 g or ≤10 g NSP/d; whereas Forslund et al. [84] and Pettersson et al. [87] focused on qualitative dietary changes favoring soluble fiber without specific amount (grams) targets. Among the protein-based interventions, bovine lactoferrin was given at 200 mg/d [49] and bovine colostrum at 0.5–1 g/kg/d [73]. Interventions started either 2 wk prior to [58,89] or at the same time as treatment [46,49,73,76], with durations extending from 3 wk [46] to as long as 24–26 mo in diet-based studies [84,87], and ≤6 mo in long-term supplementation trials [18].

#### Synbiotics

The 11 synbiotic studies were administered alongside chemoradiotherapy for cervical [50], esophageal [57,69,70], pelvic [66,77], prostate [71], and colorectal [19,77] cancers and chemotherapy for lung [17], breast [64], and pediatric cancers [56,59]. Synbiotic formulations typically combined multistrain probiotics with fiber-based prebiotics, delivered as capsules [19,56,59,64], sachets [17,71], or gels [50]. Others used liquid formulations [57,69], enteral nutrition [70], or vials [77]. Common probiotic species included *L*. *acidophilus, L. rhamnosus, L. casei, Bifidobacterium breve, B. longum, and Streptococcus thermophilus*, often delivered in commercial blends (e.g., LactoCare) and in doses ranging from 10^7^ [50] to 5 × 10^9^ CFU/d [56,59,64]. These were paired with 21 g/d [64] fructo-oligosaccharides [19,56,59,64]; 0.5–15 g/d [69,70,77] galacto-oligosaccharides [57,69,70,77]; 1.9 g/d oligofructose [17]; 4.3–8.6 g/d [71] inulin [50,71]; or 30 g/d honey [66].

Timing of synbiotic intake generally aligned with the start of cancer therapy. Five studies initiated supplementation on the first day of chemotherapy or radiotherapy [17,56,59,64,77], whereas others began 2–7 d prior [57,[69], [70], [71]]. Duration of interventions ranged from 7 d [56] to 8 wk [64].

#### Fecal microbiota transplantation

FMT was investigated in 4 studies, 3 of which targeted patients with metastatic melanoma refractory to anti–PD-1 therapy [24,25,74], and 1 study addressing gastrointestinal toxicity in metastatic renal cell carcinoma patients treated with tyrosine kinase inhibitors [61]. FMT was delivered via colonoscopy [25,61], oral capsules [74], or both [24], and used donor material from either healthy individuals [61,74] or long-term immunotherapy responders [24,25]. Only Baruch et al. [24] applied antibiotic preconditioning (vancomycin and neomycin) to deplete native microbiota prior to engraftment.

FMT administration varied in intensity. Baruch et al. [24] administered 6 cycles of FMT using both colonoscopy and capsules alongside nivolumab every 14 d over a 90-d period. Davar et al. [25] used a single colonoscopy dose along with pembrolizumab, with subsequent pembrolizumab every 3 wk (≤35 cycles or until progression/toxicity). Routy et al. [74] administered a single dose of oral capsule-based FMT (10^13^ CFU per mL of microbes) 1 wk prior to starting anti–PD-1 immunotherapy (nivolumab or pembrolizumab), given every 3–4 wk for ≤12 mo. Ianiro et al. [61] delivered a 1-time FMT dose via colonoscopy (∼57 g stool in saline) with follow-up at weeks 1, 2, 4, and 8. Apart from Routy et al. [74] who reported a dosage 10^13^ CFU per mL of microbes, no other studies indicated the concentration (CFU) of GM transferred to participants in each cycle.

In all melanoma trials, FMT was initiated shortly before or at the start of immune checkpoint blockade therapy. Baruch et al. [24] integrated FMT directly with nivolumab cycles; Davar et al. [25] initiated pembrolizumab alongside FMT; Routy et al. [74] allowed a 1-wk lead-in between capsule-based FMT and immunotherapy onset. The melanoma studies observed patients over months-long timelines (up to a year). In contrast, Ianiro et al. [61] applied FMT to previously treated patients and monitored outcomes within an 8-wk window. This was also the only study that included a controlled condition (placebo vehicle FMT, 250 mL water via colonoscopy). All other studies were uncontrolled, pre-post designs comparing outcomes against historical benchmarks for primary refractory melanoma. Specifically, Baruch et al. [24] and Davar et al. [25] enrolled patients who had previously failed anti–PD-1 therapy, with the cohort in the study by Davar et al. [25] including those unresponsive to prior anti–PD-1 monotherapy or combination regimens with CTLA-4. Routy et al. [74] enrolled only previously untreated patients initiating immunotherapy.

#### Nutritional support

Several studies reported providing dietary guidance that included the recommendation of low-fat diets alongside probiotic [51,53,75] and prebiotic supplementations [58,91]. Five studies reported use of enteral or parenteral nutrition [16,69,70,81,86], whereas 1 excluded such patients [89]. One study included nutritional counseling [46], but most (*n* = 44) did not specify any nutritional support.

#### Exclusion and inclusion criteria used in the included studies

There was not a specific and homogenous set of exclusion criteria regarding participants’ diet for the duration of the interventions. Fermented foods like yogurt were excluded in 7 studies [27,47,54,58,60,66,82] and nutritional supplements (beyond the intervention) in 6 [50,54,72,74,82,83]. Alcohol and caffeine were restricted in 2 studies [53,65].

Twenty-six studies excluded patients with chronic gastrointestinal conditions (e.g., inflammatory bowel disease, Crohn disease, ulcerative colitis, or celiac) [15,19,27,28,[46], [47], [48],[52], [53], [54],^56^,^58^,^60^,^61^,[64], [65], [66],^71^,[74], [75], [76], [77],^79^,^80^,^84^,^87^,^90^,^91^]. Several also excluded those with recent antibiotic use (from 2 wk to 3 mo prior to the study) [[15], [16], [17],^48^,^52^,^56^,^58^,^60^,^61^,^63^,^64^,^66^,^74^,^85^,^88^,^90^], and 11 studies prohibited antibiotic use during the trial [25,48,52,54,58,60,66,74,85,89,90]. Four studies excluded patients with active infections 67,68,74,88], and 8 excluded neutropenic or immunocompromised individuals 27,56,62,65,74,78,83,88], including 3 pediatric studies [56,78,88].

### Outcome reporting methods

A diverse range of outcome reporting methods were used for GIAEs and tumor response. The most commonly used system for grading GIAEs was the National Cancer Institute’s Common Terminology Criteria for Adverse Events (CTCAE), used in 22 studies across versions 2.0 to 5.0 [16,17,19,28,47,54,55,58,60,61,[65], [66], [67], [68], [69], [70],^72^,^73^,^76^,^81^,^83^,^89^]. The WHOation grading was used in 3 older studies [[51], [52], [53]]. The Bristol Stool Scale was applied in 9 studies [50,56,58,60,64,66,80,88,89], and others used in individual studies: Inflammatory Bowel Disease Questionnaire-Bowel Subset [80]; Rhodes Index of Nausea and Vomiting [64]; National Institute of Cancerology nausea and vomiting scale [50], and Radiation Therapy Oncology Group toxicity scale [76]. Quality-of-life tools were also common, with 8 studies using the European Organization for Research and Treatment of Cancer (EORTC) QLQ-C30 [17,46,58,60,84,85,87,89] and 1[17,46,58,60,84,85,87,89] and 1 using the radiotherapy-specific EORTC QLQ Proctitis module for radiotherapy patients [71]. Self-reported outcomes—via patients, parents, or physicians—were recorded in 7 studies, as standalone tools or alongside structured instruments [15,18,59,75,78,90,91]. For tumor response, 9 studies applied Response Evaluation Criteria in Solid Tumors (RECIST) [25,27,28,48,57,62,82] or immune-modified RECIST [24,28] criteria, whereas others used simplified definitions of complete or partial response based on lesion size and duration [55,86].

### Outcomes

#### Gastrointestinal adverse events

**FIGURE 2:**
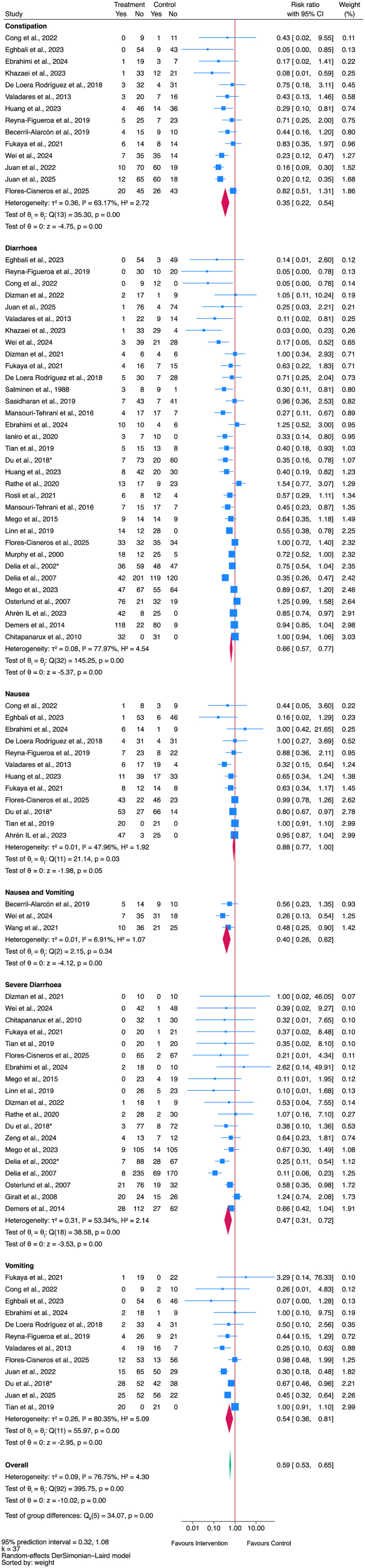
Meta-analysis showing the overall effect of probiotics, prebiotics, synbiotics, and fecal microbiota transplantation on gastrointestinal adverse events.

Based on a meta-analysis using the 37 controlled trials reporting incidence of any GIAE (3482 participants) [[15], [16], [17], [18],^27^,[45], [46], [47], [48],[50], [51], [52], [53], [54], [55], [56], [57],^60^,^61^,[63], [64], [65], [66], [67], [68],^72^,^73^,^75^,^76^,^79^,[81], [82], [83],^85^,^88^,^89^,^91^], the use of GM interventions reduced risk of GIAEs compared with control (RR: 0.59; 95% CI: 0.53, 0.65; *I*^2^: 76.8%), with high heterogeneity observed between groups and a 95% PI of 0.32–1.08 (Figure 2). There appears to be publication bias in a funnel plot asymmetry test (Supplementary Material 2), confirmed by the modified version of Egger regression test, the Harbord test (*P* = 0.020).

##### Diarrhea

Based on a subgroup meta-analysis using the 33 controlled trials reporting diarrhea incidence (3102 participants) [[15], [16], [17], [18],^27^,^45^,^47^,^48^,[50], [51], [52],[54], [55], [56], [57],^61^,[64], [65], [66], [67], [68],^72^,^73^,^75^,^76^,^82^,^83^,^85^,^88^,^89^,^91^], the use of GM interventions reduced risk of diarrhea compared with control (RR: 0.66; 95% CI: 0.57, 0.77; *I*^2^: 78.0%) (Supplementary Material 3). Based on a subgroup meta-analysis using the 19 controlled trials reporting severe diarrhea incidence (defined as ≥grade 3 diarrhea in line with National Cancer Institute’s CTCAE, the most common reporting method used by the included studies; 2216 participants) [16,17,27,47,[51], [52], [53], [54], [55],^57^,^60^,^65^,^67^,^68^,^72^,^73^,[81], [82], [83]], the use of GM interventions reduced risk of severe diarrhea compared with control (RR: 0.47; 95% CI: 0.31, 0.72; *I*^2^: 53.3%) (Supplementary Material 4).

Studies reporting diarrhea measured as duration of diarrhea (days) reported no significant reduction in the intervention group compared with control [58,78,80], whereas severity of diarrhea was significantly decreased in a synbiotic group compared with that in the control according to a self-developed study scale [19]. Synbiotics were also shown to significantly reduce diarrhea severity compared with both antibiotics [70] and probiotics [69], and no severe diarrhea was reported by Scartoni et al. [77] in a phase II study providing the synbiotic Dixentil as prophylaxis to patients undergoing pelvic radiotherapy.

Synbiotics, which combine prebiotics and probiotics, reduced diarrhea incidence (subgroup analysis, RR: 0.27, 95% CI: 0.13, 0.59) and overall GIAEs (RR: 0.36, 95% CI: 0.25, 0.52) (Supplementary Material 5), with nearly all studies reporting reductions in diarrhea [17,19,56,57,59,64,66,69,70]. In a subgroup analysis of diarrhea by intervention type, synbiotics seemed more effective than probiotics (RR: 0.27 compared with 0.70) (Supplementary Material 3).

##### Constipation

The use of GM interventions reduced risk of constipation compared with control (RR: 0.35; 95% CI: 0.22, 0.54; *I*^2^: 63.2%) (Figure 2) based on a subgroup meta-analysis using the 14 controlled trials reporting constipation incidence (1119 participants) [15,17,18,46,48,50,56,57,63,64,82,83,85,88]. One study reporting constipation by symptom score reported no statistical significance between scores in the prebiotic and placebo group [58]. Supplementary Material 6 shows results for constipation with studies stratified by type of intervention.

##### Nausea and vomiting

Risk of vomiting (RR: 0.54; 95% CI: 0.36, 0.81; *I*^2^: 80.4%) and nausea and vomiting were reduced by GM interventions compared with those by control (RR: 0.40; 95% CI: 0.26, 0.62; *I*^2^: 6.9%) (Figure 2) in subgroup meta-analyses using 12 studies reporting vomiting incidence (1112 participants) [16,18,48,50,[55], [56], [57],^63^,^82^,^83^,^85^,88] and 3[16,18,48,50,55–57,63,82,83,85,88] and 3 studies reporting nausea and vomiting incidence (221 participants) [17,46,79]. Based on 12 studies reporting nausea incidence (995 participants) [15,16,18,45,48,50,[55], [56], [57],^82^,^83^,^88^], there was insufficient evidence to suggest a reduction in nausea risk (RR: 0.88; 95% CI: 0.77, 1.00; *I*^2^: 48.0%). Two studies reported nausea and vomiting by symptom score and found no significant difference between the groups taking synbiotics or prebiotics and control groups [58,64]. Supplementary Materials 7 and 8 show results for nausea and vomiting with studies stratified by type of intervention.

#### Age

The use of GM interventions reduced risk of GIAEs overall in both adults (RR: 0.59; 95% CI: 0.53, 0.65; *I*^2^: 78.7%) (Supplementary Material 9) and children (RR: 0.61; 95% CI: 0.44, 0.85; *I*^2^: 45.8%) (Supplementary Material 10), with less heterogeneity in the 7 pediatric studies. Upon subgroup analysis, risk of diarrhea, severe diarrhea, constipation, vomiting, and nausea and vomiting were reduced in adults but not in children (Supplementary Materials 9 and 10).

#### Study design

Sensitivity analysis of RCTs (2 studies excluded) [51,55] did not change the pooled effect, only the 95% CIs: RR: 0.59; 95% CI: 0.53, 0.66; *I*^2^: 77.0% for only RCTs compared with RR: 0.59; 95% CI: 0.53, 0.65; *I*^2^: 76.8% for all study designs) (Supplementary Material 11).

### Disease response

**FIGURE 3:**
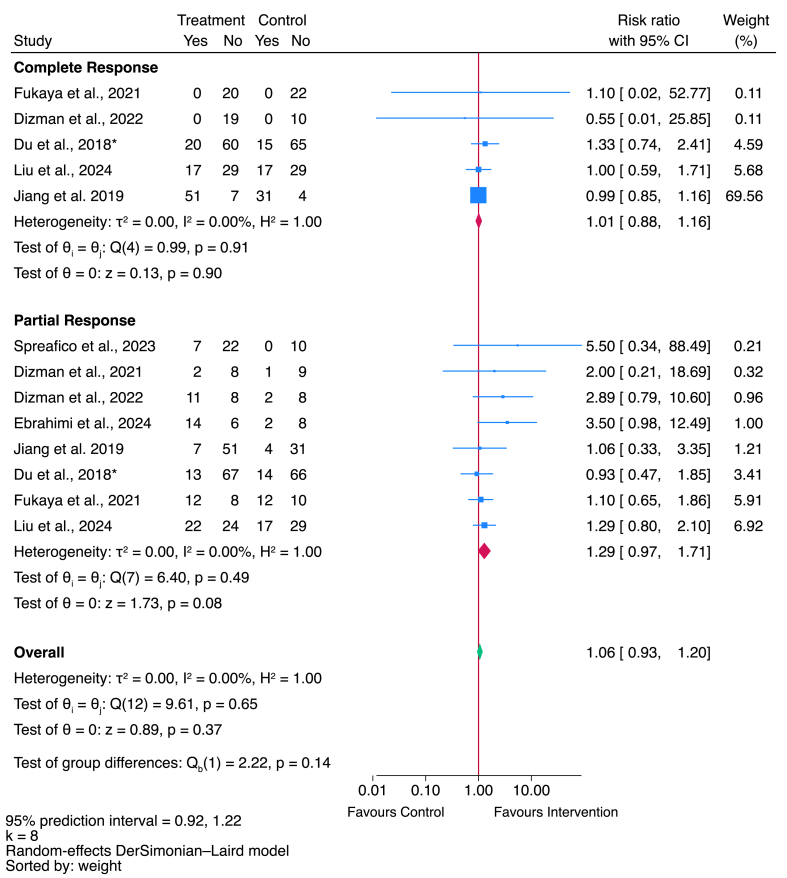
Meta-analysis showing the effects of gut microbiome interventions on objective disease response.

Eleven of the 56 studies reported the secondary outcome, ORRs [24,25,27,28,54,55,57,62,74,82,86]. ORRs in the intervention group ranged between 20% [25,54] and 100[25,54] and 100% [62], with a mean of 52.7%. Three pre-post studies using FMT observed rates of 65%, 30%, and 20% [24,25,74], with 2 of the studies using patients who were previously nonresponding to treatment [24,25]. A combination of probiotics with immunotherapy [27,28], probiotics with immunotherapy and biological therapy [82] and probiotics with chemoradiotherapy [86], increased ORRs significantly compared with control; however, the use of synbiotics and probiotics in biological therapy, chemotherapy, and radiotherapy alone did not significantly increase ORRs [54,55,57,62]. Based on a meta-analysis of the 8 controlled trials (505 participants) [27,28,54,55,57,62,82,86], there was insufficient evidence to suggest that use of GM interventions increased ORRs compared with that of control (RR: 1.06; 95% CI: 0.93, 1.20; *I*^2^: 0%; 95% PI: 0.92, 1.22) (Figure 3). A funnel plot asymmetry test (Supplementary Material 12) and the Harbord test (*P* = 0.01) showed evidence of small-study effects.

### Harmful effects

Several studies reported harmful side effects related to interventions. Probiotic-associated effects included abdominal discomfort [45], neutropenia [53], mild gastrectasia [62], and bloating with stomach pain [66]. One study noted that diarrhea could not be attributed specifically to the intervention due to chemotherapy overlap [81]. FMT-related effects included mild bloating [24], constipation [61], and grade 1–2 diarrhea, flatulence, and discomfort [74]. A high-fiber diet caused mild and self-resolving GIAEs [28]. In contrast, 21 studies reported no harmful side effects [16,47,49,52,[57], [58], [59], [60],^63^,^64^,[67], [68], [69],^71^,^73^,[76], [77], [78],^85^,^90^,^91^], whereas 26 did not specify whether harmful effects occurred [15,[17], [18], [19],^25^,^27^,^46^,^48^,^50^,^51^,[54], [55], [56],^65^,^70^,^72^,^75^,^79^,^80^,[82], [83], [84],[86], [87], [88], [89]].

### Tolerance

Scartoni et al. [77] noted that the excellent tolerance to their synbiotic is extremely important because patients undergoing chemotherapy often have olfactory/taste problems, which limit the use of micronutrient supplements. Good tolerance was reported in 9 studies [28,53,56,[58], [59], [60],^71^,^77^,^91^] based on no adverse events [[58], [59], [60],^77^,^91^], drop outs [56,58,59,71,91], and adherence to intervention (>90%) [28,53]. Of these, 3 used probiotics [28,53,60, 2[28,53,60], 2 prebiotics [58,91] and 4[58,91] and 4 synbiotics [56,59,71,77].

### Quality assessment

**FIGURE 4:**
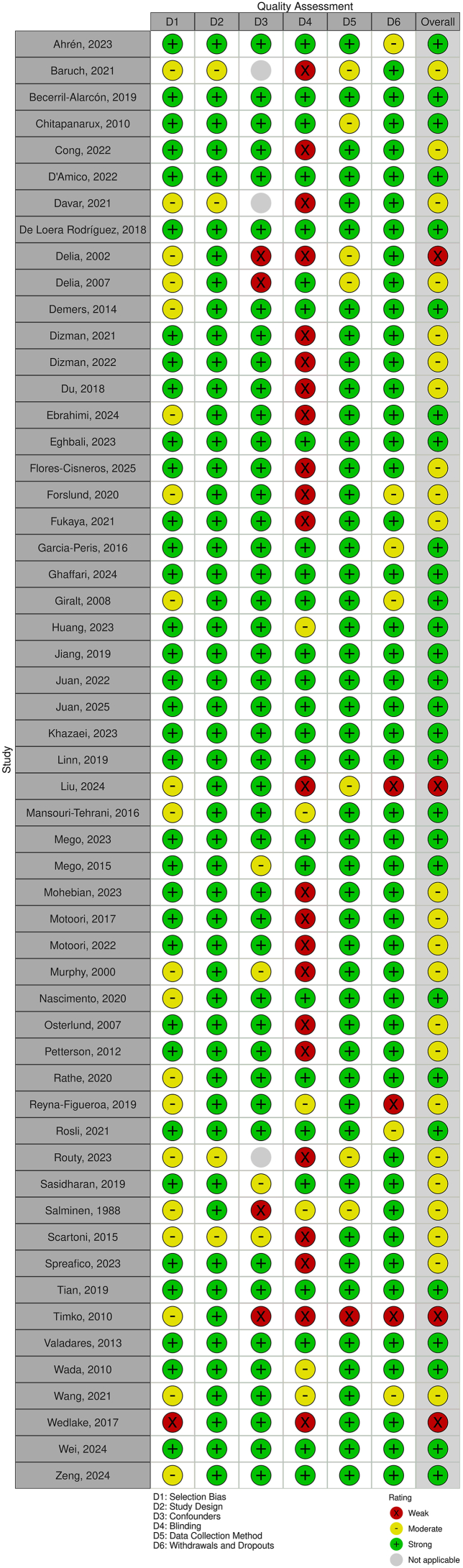
Quality assessment using the Effective Public Healthcare Panacea Project Quality Assessment tool.

The assessment of study quality is displayed in Figure 4 and Supplementary Material 13; of the 56 studies, 27 were of strong quality overall, 25 were moderate, and 4 were weak. Quality of studies was predominantly impacted by high risk of bias resulting from lack of blinding [19,24,25,27,28,48,51,54,55,57,69,70,72,74,77,80,82,83,86,87,90,91]. No trials were excluded from the systematic review based on their quality assessment rating.

## Discussion

To our knowledge, this systematic review and meta-analysis is the first to comprehensively evaluate the impact of a wide range of GM interventions—including probiotics, prebiotics, synbiotics, and FMT on GIAEs and response to treatment in patients undergoing cancer therapy. Across 56 studies (40 meta-analyzed), GM interventions were associated with a reduced risk of GIAEs (RR: 0.59; 95% CI: 0.53, 0.65), including diarrhea, constipation, nausea, and vomiting. However, there was insufficient evidence to suggest improvements in objective disease response rates (RR: 1.06; 95% CI: 0.93, 1.20), which might be explained by the high variability of cancer types and treatments included in the analysis, but could also be indicative of no meaningful benefit in cancer control.

This study extends the evidence base established by previous systematic reviews. Most prior reviews have consistently reported that GM interventions reduce the incidence of gastrointestinal toxicity in patients with cancer, especially diarrhea [[32], [33], [34],[92], [93], [94]]. However, these reviews often lacked an evaluation of disease response or focused primarily on chemotherapy or radiotherapy-induced diarrhea, with limited data on other symptoms or treatment types. This review broadens the scope by including prebiotics, synbiotics and FMT, as well as evaluating multiple cancer types and therapies in both adults and children.

### Interpretation of GIAE reduction

The beneficial effects of GM interventions on GIAEs are clinically relevant, as ≤90% of patients experience such symptoms, often leading to treatment delays or discontinuation [20,21]. The greatest evidence supports probiotics—including both multistrain blends of *Lactobacillus*, *Bifidobacterium*, and *Enterococcus* species and single-strains such as *Clostridium butyricum* and *L. rhamnosus*—administered during pelvic and colorectal radiotherapy, or chemotherapy for breast and colorectal cancers. Further evidence is needed to confirm whether this observation is due to the higher prevalence of such cancers in the population or if this is linked to the ability of such probiotics at improving mucosal immunity via lymphoid tissue [95].

Chemotherapy and radiation are the most common cancer treatments, and both disrupt the intestinal barrier, alter gut microbiota, and trigger inflammation, exacerbated by antibiotic use (frequent for patients with cancer) [21,23,96]. Probiotics restore microbial diversity, enhance short-chain fatty acid production, and support gut barrier function [11], as well as modulating gene expression related to inflammation, potentially reversing GIAEs [33,95]. These mechanisms may support the reductions in diarrhea (RR: 0.70; 95% CI: 0.5, 0.82; *n* = 20), including severe cases (RR: 0.46; 95% CI: 0.2, 0.74; n =15), constipation (RR: 0.24; 95% CI: 0.1, 0.37 *n* = 6) and vomiting (RR: 0.55; 95% CI: 0.3, 0.89; *n* = 7) observed in a subgroup analysis of probiotics (Supplementary Materials 3, 4, 6, 8, and 14).

The stronger effect was observed for constipation in patients diagnosed with breast, colorectal and pediatric cancers and treated with chemotherapy [15,63,85,88]. Indeed ∼30%–60% of patients diagnosed with breast and colorectal cancer and ALL experience constipation, likely due to treatments such as vincristine; chemotherapy agents including oxaliplatin, paclitaxel, and cyclophosphamide; as well as opioid analgesics (e.g., morphine), and antiemetics (e.g., 5-HT3 antagonists) [[15], [16], [17], [18], [19]]. This meta-analysis demonstrated a reduction in constipation with the use of probiotics strains—including *L*. *rhamnosus* GG*, B*. *longum*, *L*. *acidophilus*, and *Enterococcus faecalis*—administered at doses ranging from 0.5 × 10^5^ to 5 × 10^9^ CFU, taken 2–3 times daily during chemotherapy [15,63,85,88]. These specific strains and dosages may represent a supportive approach to help mitigate constipation in these patient populations, potentially alongside laxatives; however, further research is needed to confirm their role in treatment protocols.

Probiotics were found to reduce risk of vomiting (RR: 0.55; 95% CI: 0.34, 0.89), but there was insufficient evidence to suggest risk reduction for nausea (RR: 0.94; 95% CI: 0.86, 1.02). Nonetheless, the limited number of trials restricts definitive conclusions. Notably, the only studies showing a significant reduction in nausea involved pediatric patients with central nervous system tumors and ALL [55,88], suggesting that children may be more responsive in this context. Variation in reported outcomes—diarrhea in radiotherapy trials and nausea/vomiting in chemotherapy studies—raises the possibility that differences in efficacy reflect not only treatment type but also heterogeneity in study endpoints, measurement tools, and reporting methods. Importantly, this systematic review shows that the probiotics that significantly reduced vomiting included *L*. *rhamnosus GG, B*. *longum* combinations, and *Bacillus licheniformis*, taken 2–3 times daily during treatment and may have the potential to be used as part of treatment protocols. The lack of observed benefit on nausea in studies using probiotics such as *L*. *plantarum* HEAL9 and 299, *B*. *licheniformis, Bifidobacterium infants, L*. *acidophilus, E*. *faecalis, Bacillus cereus, L*. *rhamnosus* GG, and *C*. *butyricum* (CBM588) may be due to several factors. First, strain-specific effects are critical in probiotic efficacy, and not all strains possess the neuroactive or gut-brain axis–modulating properties needed to alleviate nausea, which is centrally mediated [12]. Second, many of these studies were designed primarily to target diarrhea or general gastrointestinal symptoms, not nausea specifically, which may affect outcome sensitivity. Third, variations in dose, timing, and duration of administration may have limited their effectiveness, as optimal delivery parameters for antinausea effects remain unclear [11,12]. Fourth, the mechanism of nausea—particularly in chemotherapy or radiotherapy—is complex and may not be sufficiently mitigated by the local gut effects of these strains, especially if they lack significant impact on inflammatory pathways or serotonergic signaling involved in emesis [97]. However, the point estimates observed in this study are similar to those for 5-HT3 antagonists compared with those of placebo among patients receiving abdominal/pelvic radiotherapy (OR for vomiting: 0.49; 95% CI: 0.33, 0.72; OR for nausea: 0.43; 95% CI: 0.26, 0.70) [98], suggesting possibly clinically relevant findings. Finally, nausea is more subjective than vomiting and very difficult for young children to describe. Therefore, although these probiotics may be effective in reducing nausea and vomiting, they require more targeted investigation.

For prebiotic studies, diarrhea reduction was the most frequently reported benefit in the systematic review [18,73,76,89,91], but there was insufficient evidence to suggest risk reduction in a subgroup analysis (Supplementary Materials 3 and 15). Overall, prebiotics reduced risk of any GIAE (RR: 0.71, 95% CI: 0.56, 0.90), but for each symptom individually, risk was not reduced (Supplementary Material 15). Qualitatively, these studies reported reductions in bloating/flatulence [84,87], watery stools [58], neutropenia [49], and improvement in irritable bowel scores [80], and the high-fiber diets improved constipation and bloating [80,83,84,87]. The role of fiber in reducing GIAEs is likely a result of its fermentation in the large intestine, which produces short-chain fatty acids, including butyrate (the main energy source for enterocytes), and thus improving gut barrier integrity and reducing local inflammation. Two synbiotic trials showed that synbiotics outperformed probiotics and antibiotics in diarrhea prevention, pointing at the synergistic effect of synbiotics, that is, prebiotics enabling the survival and effect of probiotics [69,70]. Although effects on alleviating GIAEs is promising, the variability in prebiotic type and doses used in both prebiotic and synbiotic supplements makes it difficult to attribute positive gastrointestinal effects to a specific type, dose, or combination with bacterial strains.

Likewise, although FMT showed promising results in the reduction of diarrhea compared with placebo in 1 study [61], more studies in this area are needed to understand the true effect on GIAEs and whether these vary across different cancer types, populations, and cancer treatment regimes.

### Disease response

There is currently not enough evidence to suggest that GM interventions improve objective disease response rates, despite individual studies (particularly those combining probiotics with immunotherapy or biological agents) [27,28,54,82] showing improved responses. Pre-post FMT studies showed ≥20% disease response rates with either healthy or anti–PD-1 respondent donors. The later type of studies showed ORRs of 20%, and 30% in immunotherapy-refractory melanoma patients—suggesting that this type of FMT may help overcome anti–PD-1 resistance in patients [24,25]. The GM modulates systemic immunity through its interactions with gut-associated lymphoid tissue. A diverse microbiota is associated with better immunotherapy responses [[99], [100], [101], [102]], by maintaining gut barrier integrity and preventing systemic inflammation [11,29,30].

The lack of effect may stem from several limitations: only 8 controlled trials (7 of which used probiotics, 1 synbiotics) reported ORRs, with substantial heterogeneity in cancer types, therapies, and intervention protocols. The small sample sizes are reflected in the large CIs, although removal of studies with extremely large CIs did not change the results (Supplementary Material 16). Additionally, the GM can metabolize drugs, potentially inactivating them and reducing treatment efficacy [22]. Despite limited evidence for a direct effect on ORRs, symptom relief could improve long-term outcomes by enhancing treatment adherence, reducing interruptions, and helping patients complete therapies [21,103].

### Safety

Although most studies reported no harmful effects, safety reporting was inconsistent. Mild symptoms such as bloating and discomfort were occasionally reported, and no sepsis events from probiotics were observed. Several studies excluded immunocompromised or neutropenic patients or limited intervention use to periods of immune stability, reducing risk of probiotic-related infections. This, however, would significantly limit the utility of GM interventions, and without standardized reporting, the full safety profile remains unclear.

### Pediatric implications

Seven pediatric studies, mostly in children with ALL undergoing chemotherapy, reported benefits similar to adult cohorts. All but 1 study showed reduced diarrhea with probiotics, prebiotics, or synbiotics. Some also reported improvements in constipation, nausea, and vomiting. One study found improved treatment response with probiotics [55]. Febrile neutropenia was significantly lower in 2 studies [49,78], and no serious infections were attributed to the interventions. Safety and tolerance were generally good, with no harmful effects reported in 4 of the 7 studies [49,59,73,78]. However, 3 studies did not specify whether harmful effects occurred [55,56,88]. GM interventions appear safe and effective in pediatric cancer care, particularly for managing gastrointestinal symptoms. Larger, well-controlled trials are needed to confirm benefits and guide clinical use.

### Methodological considerations and limitations

The overall heterogeneity in intervention types, study designs, and outcome reporting methods (*I*^2^: 76.8% for GIAEs; 95% PI: 0.32, 1.08) limits generalizability. The 95% PI indicates that, in some future settings, GM interventions may show little to no reduction in risk of GIAEs. Only 47% of pelvic and 56% of colorectal cancer studies were rated as high quality. Inconsistent blinding, varied control groups (placebo compared with no treatment), and differing definitions of GIAEs complicate comparisons. For example, Rosli et al. [89] found differing significance in diarrhea incidence depending on whether the CTCAE or Bristol Stool Chart was used. Inconsistent outcome reporting led to the exclusion of several studies from the meta-analysis, with 4 showing no significant reduction in diarrhea, constipation, nausea, or vomiting [58,64,78,80]. Funnel plot asymmetry tests suggested that smaller studies in this meta-analysis tended to report larger treatment effects, raising concerns about the validity of the pooled estimate. As noted by Sterne et al. [43], if study size predicts treatment effect, the applicability of the overall estimate to routine practice becomes highly uncertain, limiting the external validity of these results.

Additionally, exclusion criteria varied depending on primary outcomes, with some studies excluding participants without functional gastrointestinal tracts (e.g., patients with irritable bowel disease) and others not. Notably, most of the probiotic studies reporting a significant reduction in diarrhea risk excluded patients with chronic gastrointestinal conditions. Furthermore, key confounding factors such as dietary intake and antibiotic use were not consistently controlled across studies. This variability in study populations and confounding factors likely contributed to outcome heterogeneity, and it is highly probable that they influenced the observed effects.

### Clinical implications

Given their ability to reduce GIAEs with minimal side effects and potential for improving response rates, probiotics and synbiotics, especially the strains highlighted earlier, could be considered as adjunctive care during chemotherapy or radiotherapy in pelvic, colorectal, and pediatric cancers, with close monitoring of patients and continued research into these interventions. Routine implementation could improve patient quality of life, treatment adherence, and health care utilization. However, robust RCTs are warranted to inform clinical guidelines, particularly for prebiotics, FMT, and in immunotherapy contexts.

### Future directions

Future *trials* should prioritize:

• Standardization of probiotic strain, dosage, duration, and outcome definitions
• Studies comparing different types of prebiotics and dosages to identify those that provide desired beneficial effects without the side effects that are often attributed to these supplements (e.g., bloating)
• Standardization of antibiotic use and inclusion/exclusion of patients with gastrointestinal conditions
• Evaluation of treatment adherence and tolerance
• Evaluation of safety, not including primary outcomes such as GIAEs, but explicit reporting of harmful effects
• Controlled FMT trials, particularly in immunotherapy-refractory settings, to validate early promising results
• Longitudinal studies to assess the impact of symptom mitigation on long-term outcomes, including survival and sustained remission.
• Including GM composition outcomes to identify intervention-related shifts in microbial communities or metabolic function to elucidate potential mechanisms. This is particularly the case for colorectal cancer, where a recent meta-analysis identified key bacterial strains that play roles in development and prevention of cancer [104].

Future reviews should prioritize:

• Stratification by cancer type, treatment method, and immune status
• Inclusion of mechanistic end points (e.g., GM composition, short-chain fatty acid concentrations, and gut permeability biomarkers)
• Assessment of cost-effectiveness to guide clinical adoption, although probiotics, prebiotics, and synbiotics are relatively inexpensive, so this may only be necessary for FMT.

## Conclusion

GM interventions—particularly probiotics and synbiotics—demonstrate potential in reducing GIAEs in patients with cancer, with the strongest benefits observed during chemotherapy and radiotherapy. Synbiotics may offer enhanced efficacy through synergistic prebiotic-probiotic interactions, although prebiotics alone showed more variable outcomes. Although there is insufficient evidence in this review to indicate that GM interventions improve objective disease response rates, emerging evidence suggests probiotics and especially FMT may enhance treatment response in immunotherapy settings. However, FMT remains complex, with higher risk and resource demands, and currently lacks robust controlled evidence. Future research should prioritize well-powered, standardized trials across diverse cancer types and therapies to confirm efficacy, clarify mechanisms, and guide integration of microbiome-based strategies into personalized cancer care.

## Author contributions

The authors’ responsibilities were as follows—CM, RRI, LT: designed the study; CM, RL: conducted the search, screening, risk, and data extraction; RRI, LT: solved any disagreements; CM: performed all meta-analysis; CM, RRI, LT: wrote the manuscript; RL, CFL, MFHB, JFP-G, RRI, LT: critically revised the manuscript for important intellectual content; LT and RRI: were responsible for overall study supervision; and all authors: read and approved the final manuscript.

## Data availability

Data described in the manuscript, code book, and analytic code will be made publicly and freely available without restriction at Open Research Exeter (ORE; https://ore.exeter.ac.uk/repository/).

## Funding

The authors reported no funding received for this study.

## Conflict of interest

The authors report no conflicts of interest.
