# Supplementary material for: Interventions Targeting the Gut Microbiome to Improve Cancer Treatment Outcomes and Their Gastrointestinal Side Effects: A Systematic Review and Meta-analysis
Source: J Nutr. 2025 Dec 29;156(2):101300. doi: 10.1016/j.tjnut.2025.101300 (PMC12975354; doi:10.1016/j.tjnut.2025.101300)
Supplement: Multimedia component 1 [file mmc1.docx]

Supplementary Material for:

**Interventions targeting the gut microbiome to improve cancer treatment outcomes and their gastrointestinal side effects: a systematic review and meta-analysis**

Cecilia Morel et al.

**Table of Contents**

| **Supplementary Material 1.** Search Strategy | 3-4 |
| --- | --- |
| **Supplementary Material 2.** Gastrointestinal Adverse Events Funnel Plot | 5 |
| **Supplementary Material 3**. Meta-analysis showing the effects of pre-, pro- and synbiotics and Faecal Microbiota Transplantation on diarrhoea occurrence | 6 |
| **Supplementary Material 4**. Meta-analysis showing the effects of pre-, pro- and synbiotics on severe diarrhoea occurrence | 7 |
| **Supplementary Material 5**. Meta-analysis showing the effects of synbiotics on Gastrointestinal Adverse Events | 8 |
| **Supplementary Material 6**. Meta-analysis showing the effects of pre-, pro- and synbiotics on constipation occurrence | 9 |
| **Supplementary Material 7.** Meta-analysis showing the effects of pre-, pro- and synbiotics on nausea occurrence | 10 |
| **Supplementary Material 8**. Meta-analysis showing the effects of pre-, pro- and synbiotics on vomiting occurrence | 11 |
| **Supplementary Material 9**. Sensitivity analysis showing the effects of gut microbiome interventions on Gastrointestinal Adverse Events in adults | 12 |
| **Supplementary Material 10**. Sensitivity analysis showing the effects of gut microbiome interventions on Gastrointestinal Adverse Events in children | 13 |
| **Supplementary Material 11**. Sensitivity analysis showing the effects of gut microbiome interventions on Gastrointestinal Adverse Events restricted to randomised controlled trials | 14 |
| **Supplementary Material 12.** Overall Response Funnel Plot | 15 |
| **Supplementary Material 13.** Quality Assessment using The Effective Public Healthcare Panacea Project Quality Assessment tool | 15 |
| **Supplementary Material 14.** Meta-analysis showing the effects of probiotics on Gastrointestinal Adverse Events | 16 |
| **Supplementary Material 15.** Meta-analysis showing the effects of prebiotics on Gastrointestinal Adverse Events | 17 |
| **Supplementary Material 16.** Sensitivity analysis showing the effects of gut microbiome interventions on overall response, excluding studies with large confidence intervals | 18 |

**Supplementary Material 1.** Search Strategy

"cancer*" OR "neoplasm" OR "tumour*" OR "tumor*" OR “tumoural” OR “tumourous” or “cancerated” OR “cancerous” OR “malignant” OR "malignancy" OR "malign*" OR "carcinoma" OR "melanoma" OR "cyst*" OR "neurofibroma*” OR “astrocytomas” OR “acute lymphoblastic leukemia” OR “ALL” OR “acute myeloid leukemia” OR “leukemia” OR “leukaemia” OR “AML” OR “carcinoma” OR “lymphoma” OR “sarcoma” OR “osteosarcoma” OR “malignant fibrous histiocytoma” OR “medulloblastoma” OR “cholangiocarcinoma” OR “chordoma” OR “CLL” OR “chronic myelogenous leukemia” OR “CML” OR “craniopharyngioma” OR “DCIS” OR “ependymoma” OR “esthesioneuroblastoma” OR “retinoblastoma” OR “GIST” OR “gestational trophoblastic disease” OR “langerhans cell histiocytosis” OR “pleuropulmonary blastoma” OR “multiple endocrine neoplasia syndromes” OR “multiple myeloma” OR “mycosis fungoides” OR “myelodysplastic syndrome” OR “neuroblastoma” OR “papillomatosis” OR “paraganglioma” OR “pheochromocytoma” OR “rhabdomyosarcoma” OR “sézary syndrome”

(MeSH Terms if applicable: “neoplasms”)

**AND**

“chemotherapy” OR “radiotherapy” OR “cisplatin*” OR “therap*” OR “adjuvant chemotherapy” OR "neo-adjuvant chemotherapy" OR “drug therapy” OR “pharmacologic therapy” OR “medicine treatment” OR “radiation therapy” OR “chemoradiotherapy” OR “radiotherapy” OR “radiother*” OR “neoadjuvant therapy” OR “neoadjuvant*” OR "immunotherapy" OR "immune checkpoint inhibitors" OR "immunotherap*" OR "biologic therap*" OR "biotherap*" OR "chimeric antigen receptor therap*" OR "CAR T-cell therap*" OR "immune checkpoint inhibit*" OR "immune checkpoint block*" OR "PD-L1 inhibit*" OR "PDL1 inhibit*" OR "programmed death ligand 1 inhibit*" OR "CTLA-4 inhibit*" OR "cytotoxic T-lymphocyte-associated protein 4 inhibit*" OR "PD-1 inhibit*” OR "anti PD 1" OR "anti-PD-1" OR "anti PD-1" OR "anti PD-L1" OR "anti-CTLA-4" OR "anti CTLA 4" OR "Pembrolizumab" OR "Keytruda" OR "Opdivo" OR "Nivolumab" OR "Ipilimumab" OR "atezolizumab" OR "avelumab" OR "durvalumab" OR "lambrolizumab" OR "pidilizumab" OR "tremelimumab" OR “ICI” OR “CPI” OR “checkpoint blockade” OR “ICB” OR “inhibitor*”

(MeSH Terms if applicable: “radiotherapy” OR “immunotherapy”)

**AND**

"gut microbio*" OR "gastrointestinal" OR "gastrointestinal microbio*" OR "gut" OR "gastrointestinal microbial community" OR "gastric microbiome" OR "intestinal bacter*" OR "enteric bacteria" OR "microbio*" OR "probiot*" OR "prebio*" OR "synbio*" OR "metagenome*" OR "flora" OR "microflora" OR "lactobacill*" OR "lactobacteri*" OR "bifidobacter*" OR "lactococc*" OR "saccharomyce*" OR "streptococcus thermophilus" OR "lactic acid bacteri*" OR "bacillus subtilis" OR "enterococcus" OR "firmicute*" OR "bacteroid*" OR "ruminococcac*" OR "akkermansia muciniphila" OR "Verrucomicrobiot*" OR "SCFA*" OR "short chain fatty acid*" OR "actinobacillus" OR "Proteobacteri*" OR "short-chain fatty acid*" OR "butyrate" OR "acetate" OR "propionate" OR "Faecalibacteri*" OR "Clostridial*" OR "Escherichia coli" OR "Akkermansiaceae" OR "Akkermansia*" OR "muciniphila" OR "FMT" OR "feces" OR "fecal" OR "fecal microbiota transplant*" OR "faecal microbiota transplant*" OR "donor" OR "infus*” OR "enema*" OR "stool*" OR "fecal bacteriotherap*" OR "faecal" OR "microbial consortium" OR "MET" OR "microbial ecosystem therapeutic*" OR "CBM588” OR "CBM-588" OR "SER401" OR "SER-401" OR "buty*" OR "fibre" OR "dietary fibre" OR "diet" OR "MET4" OR "clostridi*" OR "supplement*" OR "bacter*"

(MeSH Terms if applicable: "gastrointestinal microbiome" OR "probiotics" OR "prebiotics” OR "synbiotics” OR “faecal microbiota transplantation”)

**Supplementary Material 2.** Gastrointestinal Adverse Events Funnel Plot

**Supplementary Material 3**. Meta-analysis showing the effects of pre-, pro- and synbiotics and Faecal Microbiota Transplantation on diarrhoea occurrence

**Supplementary Material 4**. Meta-analysis showing the effects of pre-, pro- and synbiotics on severe diarrhoea occurrence

**Supplementary Material 5**. Meta-analysis showing the effects of synbiotics on Gastrointestinal Adverse Events

**Supplementary Material 6**. Meta-analysis showing the effects of pre-, pro- and synbiotics on constipation occurrence

**Supplementary Material 7.** Meta-analysis showing the effects of pre-, pro- and synbiotics on nausea occurrence

**Supplementary Material 8**. Meta-analysis showing the effects of pre-, pro- and synbiotics on vomiting occurrence


**Supplementary Material 9**. Sensitivity analysis showing the effects of gut microbiome interventions on Gastrointestinal Adverse Events in adults

**Supplementary Material 10**. Sensitivity analysis showing the effects of gut microbiome interventions on Gastrointestinal Adverse Events in children

**Supplementary Material 11**. Sensitivity analysis showing the effects of gut microbiome interventions on Gastrointestinal Adverse Events restricted to randomised controlled trials

**Supplementary Material 12.** Overall Response Funnel Plot

**Supplementary Material 13.** Quality Assessment using The Effective Public Healthcare Panacea Project Quality Assessment tool


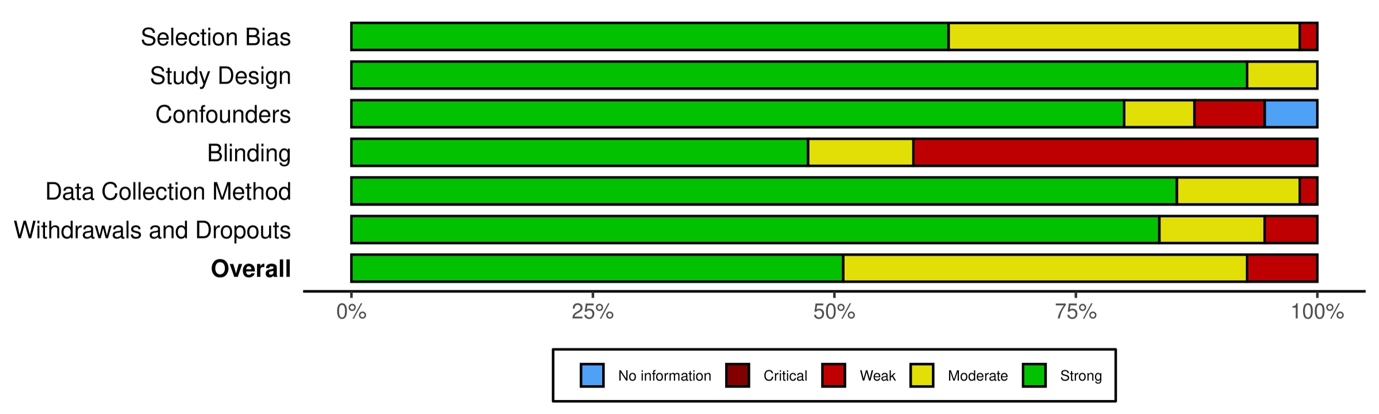

**Supplementary Material 14.** Meta-analysis showing the effects of probiotics on Gastrointestinal Adverse Events

**Supplementary Material 15.** Meta-analysis showing the effects of prebiotics on Gastrointestinal Adverse Events

**Supplementary Material 16.** Sensitivity analysis showing the effects of gut microbiome interventions on overall response, excluding studies with large confidence intervals
